# Promoting Alkane Binding: Crystallization of a Cationic Manganese(I)‐Pentane *σ*‐Complex from Solution

**DOI:** 10.1002/anie.202507494

**Published:** 2025-05-27

**Authors:** Malte Sellin, James D. Watson, Julia Fischer, Graham E. Ball, Leslie D. Field, Ingo Krossing

**Affiliations:** ^1^ Institut für Anorganische und Analytische Chemie and Freiburg Materials Research Center FMF Albert‐Ludwigs‐Universität Freiburg Albertstr. 21 79104 Freiburg Germany; ^2^ School of Chemistry University of New South Wales Sydney NSW 2052 Australia

**Keywords:** Alkane activation, Alkanes, C─H activation, Crystallization, Low‐Temperature NMR, Sigma‐complex

## Abstract

Transition metal–alkane *σ*‐complexes are key intermediates in C–H activation and, until now, analysis of these species has been restricted to either the solution OR the solid state. Here we present a synthetic methodology that converts Mn_2_(CO)_10_ in a noncoordinating solvent environment to [Mn(CO)_5_]^+^ at room temperature, and this species binds *n*‐pentane as the strongest interacting ligand available. Three isomers of the *n*‐pentane *σ*‐complexes were studied in detail by solution NMR‐spectroscopy at low temperature. Two isomers of [Mn(CO)_5_(*n*‐pentane)]^+^ crystallize from solution at room temperature and their structures were determined by single crystal X‐ray crystallography. The oxidation of metal complex dimers in solution to generate reactive metal cations, which bind alkanes as the strongest available ligand, is a new and probably general approach to generate metal‐alkane *σ*‐complexes.

## Graphical abstract

Oxidation of Mn₂(CO)₁₀ under *pseudo gas‐phase conditions* generates the weakly coordinated 16 valence electron complex [Mn(CO)₅]⁺, which binds *n*‐pentane as the strongest ligand available in this system. Three isomers were characterized by low‐temperature NMR and X‐ray crystallography. The oxidation of metal complex dimers is a new route to generate reactive metal–alkane *σ*‐complexes.

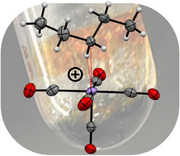

## Introduction

Selective activation of C─H bonds in saturated hydrocarbons is a chemical transformation which is highly coveted by academia and industry alike. This seemingly simple process has the potential to transition hydrocarbons from being mainly combustible fuels to synthetically useful feedstocks, unlocking enormous environmental, financial, and broad economic benefits. And, despite substantial progress in this field, this process has not yet been harnessed to its full potential. Selective activation of alkanes remains challenging.^[^ ^1^, ^2^, ^3^, ^4^, ^5^, ^6^, ^7^, ^8^, ^9^ ^]^

Thus, alkanes are exceptionally difficult substrates on which to perform chemical reactions. Comprised solely of nonpolar C─H and C─C bonds, alkanes have a very poor ability to donate electrons to electrophilic species or accept electrons from nucleophiles. The C─C and C─H bonds in alkanes have large bond dissociation energies (BDEs) for homolytic cleavage, typically between 330–380 kJ mol^−1^ and 380–440 kJ mol^−1^, respectively.^[^ ^10^ ^]^ The large BDE implies that it takes a significant amount of energy to break the C─C and/or C─H bonds in alkanes.^[^ ^11^ ^]^ Nevertheless, each C─H and C─C *σ*‐bond in an alkane has an associated pair of electrons, and there are now a number of different transition metal complexes which are sufficiently electrophilic, and have an appropriate electronic configuration, to accept electron density from *σ*‐bonds in alkanes to form metal‐alkane *σ*‐complexes where an alkane C─H bond binds to the metal center via 3‐center‐2‐electron interaction.^[^ ^12^, ^13^, ^14^, ^15^, ^16^, ^17^ ^]^ This interaction already reduces the activation barrier of the C─H bond versus M─C / M─H bond formation down to ca. 80 kJ mol^−1^.

**Figure 1:**
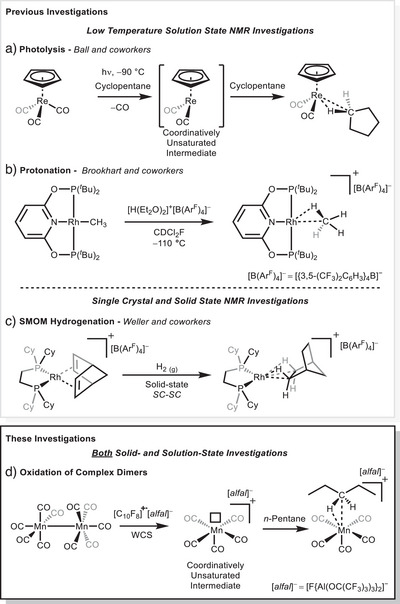
a) Photodissociation of CO from [(*η* ^5^‐Cp)Re(CO)_3_] to generate the unobserved reactive intermediates which subsequently reacts with the cyclopentane solvent to form the cyclopentane complex [*η* ^5^‐CpRe(CO)_2_(*c*‐pentane)]; b) protonation of a [(PONOP)Rh(CH_3_)] (1) (PONOP  =  *κ* ^3^‐NC_5_H_3_‐2,6‐(OP^t^Bu_2_)_2_) complex with [H(Et_2_O)_2_]^+^[B(Ar^F^)_4_]^−^ (Ar^F^  =  3,5‐(CF_3_)_2_C_6_H_3_) in CDCl_2_F at −110 °C to form the rhodium methane complex [(PONOP)Rh(CH_4_)]^+^[B(Ar^F^)_4_]^−^; c) hydrogenation of [Rh(Cy_2_PCH_2_CH_2_PCy_2_)(NBD)]^+^[B(Ar^F^)_4_]^−^ (NBD  = norbornadiene) in the solid state to effect the single crystal to single crystal (SC‐SC) formation of the alkane *σ*‐complex [Rh(Cy_2_PCH_2_CH_2_PCy_2_)(NBA)]^+^[B(Ar^F^)_4_]^−^ (NBA  =  norbornane); and d) formation of [Mn(CO)_5_(*n*‐pentane)]^+^ by oxidation of Mn_2_(CO)_10_ with [C_10_F_8_]^+·^[*alfal*]^−^ ([*alfal*]^−^  =  [F{Al(OC(CF_3_)_3_)_3_}_2_]^−^ = [(R^F^O)_3_Al‐F‐Al(OR^F^)_3_]^−^; R^F^ = C(CF_3_)_3_), in a weakly coordinating solvent.

Such alkane *σ*‐complexes have been the subject of intense investigation since the 1970′s, but their high reactivity and challenging synthesis has limited the number of in‐depth investigations into these species. Aside from the archetypal photochemical synthetic route, where UV irradiation ejects a ligand from an appropriate precursor to generate a reactive, coordinatively unsaturated species that can bind alkanes as incoming ligands, only the synthetic strategies developed by Brookhart and Weller (and deployed by several others) have been demonstrated to form alkane *σ*‐complexes.^[^ ^17^, ^18^, ^19^, ^20^ ^]^ The elegant synthetic protocols of Weller and Brookhart strategically preassemble the alkane on the metal center and then generate a formally “bound” alkane by in‐situ metal alkyl‐protonation in solution at low temperatures or by hydrogenation of an alkene complex in the solid state (Figure 1b,c).

In all of these systems, the metal acceptor and the alkane precursor are chemically bound **before** the alkane is formed close to the metal center. Until now, a true “binding” of alkanes to metal centers (rather than assembling them on a metal fragment) has been limited to photochemical techniques (Figure 1a). Many alkane *σ*‐complexes, that have been well characterized in solution using NMR and IR spectroscopy, were synthesized by photoejection of a labile ligand in the presence of an alkane. However, using this (or any other) technique, there have been no reports where the alkane *σ*‐complex was isolated and crystallized from solution, most likely due to the presence of the photoejected ligand that quickly rebinds when irradiation is removed. Other structures, where alkane C─H bonds were located close to metal centers like the uranium‐alkane complexes by Meyer et al., likely formed in large part due to host–guest and dispersion interactions.^[^ ^21^, ^22^, ^23^, ^24^, ^25^, ^26^ ^]^ Weller and coworkers have prepared the only examples of crystallographically characterized alkane *σ*‐complexes by solid‐state molecular organometallic (SMOM, (Figure 1c) synthesis. SMOM provided access to more than 11 examples of alkane *σ*‐complexes in the solid‐state.^[^ ^12^, ^19^, ^27^, ^28^, ^29^, ^30^, ^31^, ^32^, ^33^ ^]^ Despite their relative stability in the solid state, the complexes synthesized by Weller and coworkers, have not been observed in solution to date. Attempts have been made to analyze the Weller‐type alkane *σ*‐complexes using solution techniques, but, when dissolved, the alkane *σ*‐complexes decompose rapidly, forming agostic and solvent complexes, even when dissolved in very weakly coordinating solvents at very low temperatures.^[^ ^34^ ^]^

Until now, metal‐alkane *σ*‐complexes have only been observed in either solution **OR** the solid state. Thus, the development of a synthetic strategy allowing for both is a challenge addressed with this work.

The basis for our concept emerged from a key trend notable from solution investigations into alkane *σ*‐complexes: cationic metal fragments tend to bind alkanes more tightly than their neutral, isoelectronic analogues.^[^ ^35^ ^]^ Computational investigations by Head–Gordon and coworkers predicted this trend some years before the report into the cationic complex [(*η* ^6^‐HEB)Re(CO)_2_(alkane)]^+^ (HEB = hexaethylbenzene, alkane  = *n*‐C_5_H_12_ and *c*‐C_5_H_10_).^[^ ^35^, ^36^, ^37^ ^]^ Yet, it was not until this investigation that the theoretical discussion was supported by experimental evidence.

**Figure 2:**
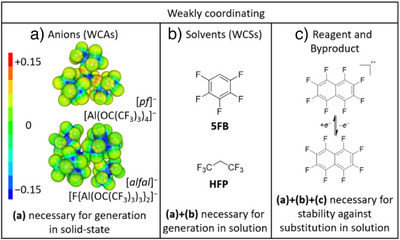
The three necessary factors for the generation of a cationic σ‐alkane complex in solution stable against substitution. a) Anions electrostatic potentials plotted on the isodensity surfaces (0.025 e^−^ B^−3^) of the WCAs [*pf*]^–^ and [*alfal*]^−^ at the B3LYP/def2‐TZVPP level of theory ([*pf*]^−^  =  [Al(OR^F^)_4_]^−^; [*alfal*]^−^ = [(R^F^O)_3_Al‐F‐Al(OR^F^)_3_]^−^; R^F^ = C(CF_3_)_3_). b) Weakly coordinating, but polar solvents. c) Weakly coordinating reagents and by‐products.

Critical to the success of the solution investigations have been the weakly coordinating anions (WCAs) and weakly coordinating solvents (WCSs) that together generate *pseudo gas‐phase conditions*.^[^ ^38^ ^]^ In particular, the perfluorinated alkoxyaluminate anions [*pf*]^−^ and [*alfal*]^−^ ([*pf*]^−^  =  [Al(OR^F^)_4_]^−^; [*alfal*]^−^ = [(R^F^O)_3_Al‐F‐Al(OR^F^)_3_]^−^; R^F^ = C(CF_3_)_3_), in combination with highly fluorinated solvents like 1,1,1,3,3,3‐hexafluoropropane (HFP), 1,2,3,4‐tetrafluorobenzene (4FB) or pentafluorobenzene (5FB) lead to success (Figure 2a,b).^[^ ^39^ ^]^ As we have discussed previously,^[^ ^35^, ^40^ ^]^ the unique properties of the fluorine atoms in these highly fluorinated anions and solvents molecules make them extremely reluctant electron pair donors. Consequently, these molecules provide an extremely weakly coordinating environment where the C─H bonds of an added alkane become the best ligands in solution.

Overall, the three mandatory factors for the generation of a cationic alkane σ‐complex in solution which is stable against substitution, are illustrated in Figure 2. Omission of any one of these components leads to side‐reactions and results in degradation of the alkane σ‐complex.

Oxidation has previously been used to cleave metal–metal bonds in transition metal carbonyl clusters to form mono‐ and multimetallic complexes. However, earlier work utilized [AsF_6_]^−^ and related WCAs, resulting in tight ion pairs like [(OC)_5_Mn(F−AsF_5_)], when no stronger ligand was available.^[^ ^41^ ^]^ Alkanes are ligands too weak to compete with the [AsF_6_]^−^ anion. Switching to the even better WCAs [*pf*]^−^ and [*alfal*]^−^, this pitfall is overcome. Similarly important, our group has developed cations, which serve as both powerful and process‐selective de‐electronators (one‐electron oxidants), but neither as Lewis acids, nor as competing Lewis bases or ligands.^[^ ^39^, ^42^, ^43^, ^44^ ^]^ These de‐electronators are typically radical cations of perhalogenated multicyclic arenes. Perhalogenation ensures a high potential of the radical cation, the weak basicity and weak nucleophilicity of the resulting neutral arene after reduction, and the absence of positively charged leaving groups (Figure 2c). The de‐electronator salt used herein is the [*alfal*]^−^ salt of the perfluoronaphthalene radical cation [C_10_F_8_]^+·^[*alfal*]^−^ with a solution de‐electronation potential of +2.00 V versus Fc^+/0^ (Fc = Fe(*η* ^5^‐C_5_H_5_)_2_) in 4FB.^[^ ^44^ ^]^ This highly potent, but essentially noncoordinating de‐electronator with an excellent WCA is used in a weakly coordinating solvent in reactions to produce cationic σ‐alkane complexes in solution stable against substitution and which are suitable for analysis by both, solution‐state and solid‐state techniques (Figure 2a–c).

## Results and Discussion

### Binding *n*‐Pentane to a Manganese Center

Prior to now, there have been investigations into the protonation of [Mn(CO)_5_(CH_3_)] with strong acids to produce methane and [Mn(CO)_5_]^+^.^[^ ^45^, ^46^ ^]^ Gas phase collision‐induced dissociation mass‐spectrometry experiments indicate that the cationic *σ*‐complex [Mn(CO)_5_(CH_4_)]^+^ likely forms prior to methane dissociation but these analyses provide little detail on the structure or binding interaction of methane to the [Mn(CO)_5_]^+^ fragment.^[^ ^47^ ^]^ There have also been several NMR and IR spectroscopic investigations into the neutral manganese alkane complexes [(*η* ^5^‐Cp)Mn(CO)_2_(alkane)] (where alkane = ethane, propane, butane, cyclopentane, and isopentane).^[^ ^48^, ^49^ ^]^ These species are generated by the photo ejection of a single carbonyl ligand from [*η* ^5^‐CpMn(CO)_3_] suspended in very cold (−138 °C) solutions of neat alkane.

Here we present the solution and solid‐state characterization of a manganese *n*‐pentane complex synthesized by the oxidation of Mn_2_(CO)_10_ in the presence of *n*‐pentane in HFP or pentafluorobenzene (5FB).

**Figure 3:**
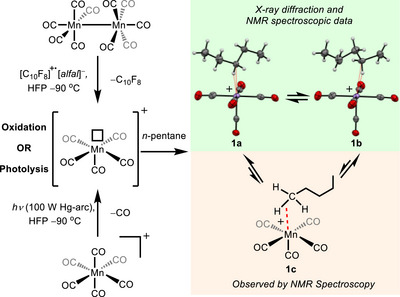
Formation of [Mn(CO)_5_(*n*‐pentane)]^+^ by oxidation of Mn_2_(CO)_10_ in weakly coordinating solvent or by photolysis of [Mn(CO)_6_]^+^ at low temperatures in a weakly coordinating solvent. [*alfal*]^−^ anions have been omitted from the complexes for clarity.

When 2 equivs of [C_10_F_8_]^+·^[*alfal*]^−^ and Mn_2_(CO)_10_ are combined in a weakly coordinating solvent HFP or 5FB, the de‐electronator removes the electrons involved in the manganese–manganese bond to form the reactive, coordinatively unsaturated complex, [Mn(CO)_5_]^+^[*alfal*]^−^ (not observed) and this species reacts with *n*‐pentane to form the *σ*‐complex [Mn(CO)_5_(*n*‐pentane)]^+^[*alfal*]^−^ (**1**) (Figure 3). The formation of **1** by oxidation was corroborated by also generating this species photochemically by photolysis (100 W, Hg‐arc lamp) of [Mn(CO)_6_]^+^[*alfal*]^−^ in the presence of *n*‐pentane. Oxidation of Mn_2_(CO)_10_ with [C_10_F_8_]^+·^[*alfal*]^−^ in the presence of *n*‐pentane reproducibly yields **1** and avoids the inherent back‐reaction with CO, which occurs if [Mn(CO)_5_]^+^ is generated photochemically from [Mn(CO)_6_]^+^ (Figure 3). Thus, when [Mn(CO)_5_(*n*‐pentane)]^+^ is generated photochemically, the signals for the pentane complexes rapidly disappear over a matter of minutes at −90 °C when the sample is not under constant irradiation.

### Solid‐State Analysis of [Mn(CO_5_)(*n*‐Pentane)]^+^

**Figure 4:**
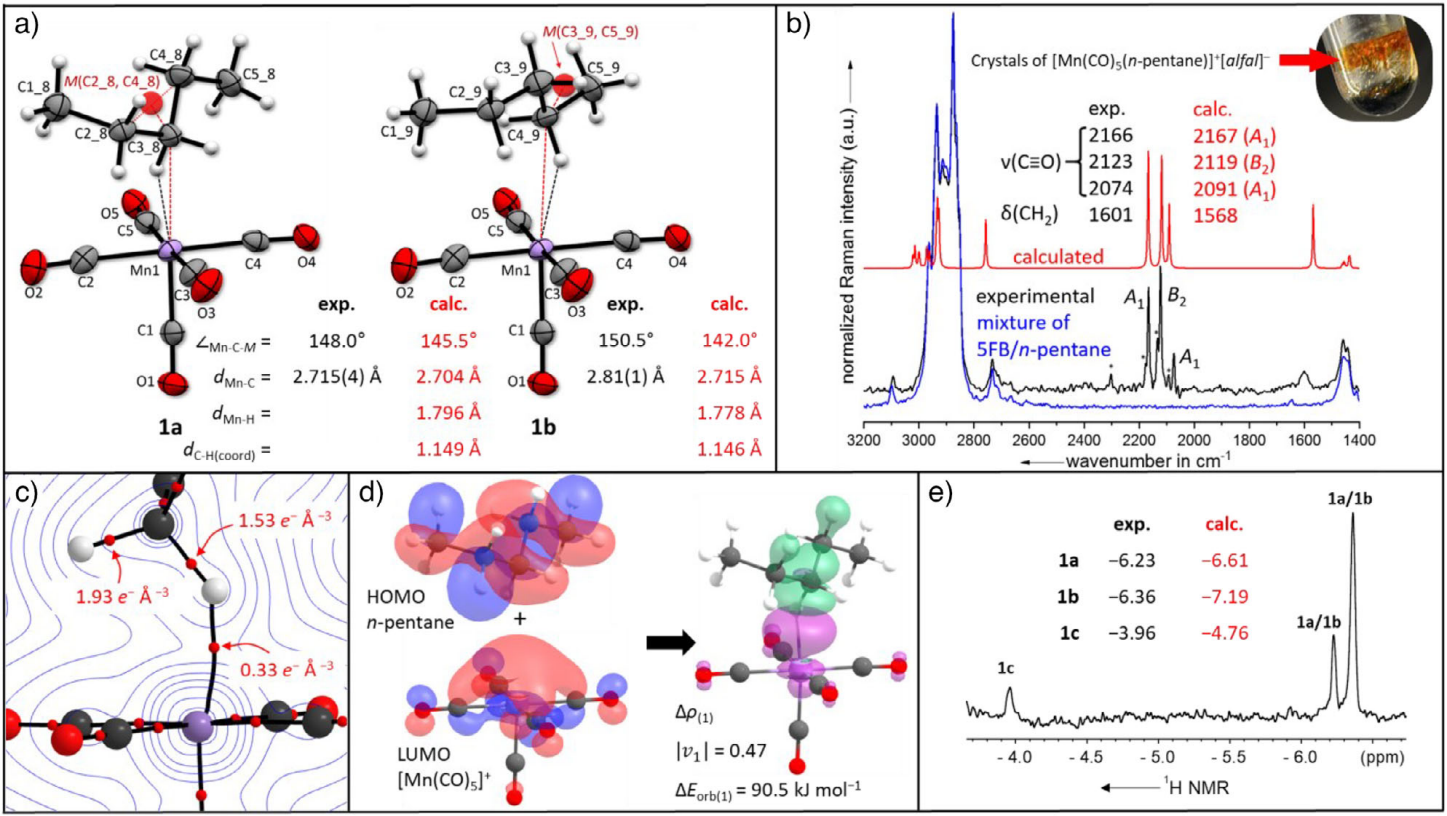
a) Molecular structures of the two isomers of [Mn(CO)_5_(*n*‐pentane)]^+^ in (disordered) [Mn(CO)_5_(*n*‐pentane)]^+^[*pf*]^−^, 1a and 1b. Mid points *M* between the two neighboring carbons atoms attached to the bound carbon atom are indicated by red spheres. Ellipsoids shown at 50% probability. b) Experimental Raman spectrum of crystalline [Mn(CO)_5_(*n*‐pentane)]^+^[*alfal*]^−^ in a mixture of *n*‐pentane and 5FB (black line) in comparison with the blank *n*‐pentane/5FB solvent mixture (blue line) and the calculated Raman spectrum of [Mn(CO)_5_(*n*‐pentane)]^+^ (red line) at the B3LYP(D3BJ)/def2‐TZVPP level of theory scaled by 0.968 according to Duncan et al. Mulliken symbols of the carbonyl vibrations are given with respect to the [Mn(CO)_5_]^+^ fragment only. Asterisks (*) denote the bands coming from a minor impurity of [Mn(CO)_5_(N_2_)]^+^[*alfal*]^−^ (see Supporting Information). c) Relief map of the electron density *ρ* (blue lines) in the Mn−H−C plane calculated at the B3LYP(D3BJ)/def2‐TZVPP level of theory. Bond critical points depicted as red dots. d) Isodensity surfaces of the HOMO of *n*‐pentane and the LUMO of [Mn(CO)_5_]^+^ (0.3 e^−^ Å^−3^) and the isodensity surface of the deformation density Δρ_(1)_ (0.01 e^−^ Å^−3^). Electron flow green → purple. Eigenvalue | ***ν***_1_| gives the sum of the migrated charge in e^−^. e) An expansion of the high field region of the 700 MHz ^1^H NMR spectrum of the three isomers of [Mn(CO)_5_(*n*‐pentane)]^+^ in a solution of HFP at −90 °C.

Yellow‐orange crystals of [Mn(CO)_5_(*n*‐pentane)]^+^[*alfal*]^−^ suitable for single crystal x‐ray diffraction were grown by layering the reaction mixture, [C_10_F_8_]^+·^[*alfal*]^−^ and Mn_2_(CO)_10_ in 5FB, with *n*‐pentane and allowing the solution to slowly diffuse over multiple days at 0 °C. [Mn(CO)_5_(*n*‐pentane)]^+^[WCA]^−^ crystallizes as two distinct isomers, where *n*‐pentane is bound at the C2 (**1b**) **or** C3 (**1a**) sites (Figure 4a) but preferentially crystallizes as the C3‐bound isomer **1a**. During our investigations, the salt [Mn(CO)_5_(*n*‐pentane)]^+^[*pf*]^−^ was also crystallized. Note, that the [*pf*]^−^ salt could not be synthesized in bulk purity. Still, the single crystal x‐ray data for [Mn(CO)_5_(*n*‐pentane)]^+^[*pf*]^−^ is of higher quality than that collected for [Mn(CO)_5_(*n*‐pentane)]^+^[*alfal*]^−^ and consequently it is this data set discussed below.

In the solid‐state, the fragment [Mn(CO)_5_]^+^ in all [Mn(CO)_5_(*n*‐pentane)]^+^[WCA]^−^ moieties is almost a perfect square‐based pyramid (*C*_4v_ symmetry), where the carbonyl ligands are at nearly 90° or 180° angles to one another (Figure 4a). The Mn─C(─H) distance in **1a** is 2.715(4) Å comparable to M─C(─H) bond distances that have been observed in other metal alkane complexes.^[^ ^33^, ^34^ ^]^ As the protons were not refined freely, the geometry of the binding site was indirectly determined by the angle between the manganese atom, the coordinating carbon atom and the midpoint *M* of the two neighboring carbons (∠_Mn–C–_ *_M_*). In principle, this angle could range from 180°, symmetrical bonding over two C─H bonds, to ca. 120° in a linear Mn,H,C arrangement. In the case of the [Mn(CO)_5_(*n*‐pentane)]^+^ complex, this angle, with both the [*pf*]^−^ and [*alfal*]^−^ anions, is within the range of 147°–151°. This distortion is a strong indication, that the two geminal protons have significantly different distances to the manganese atom, validating the binding mode over only one C─H bond.

In the solid‐state structures of **1a** and **1b**, the pentane is present in conformations containing gauche interactions rather than the all‐anti conformation. The experimentally determined Mn─C distances and the∠_Mn–C–_ *_M_* angle match the values calculated at the MN15/def2‐TZVPP level. The measured Mn─C distance in **1a** (2.715(4) Å) is comparable with that of the DFT optimized structure of **1a** (2.704 Å). However, the calculated Mn─C distance of **1b** (2.715 Å) is somewhat shorter than the experimental distance 2.81(1) Å (Figure 4a). Similarly, the C─Mn─CO_ax_ angle calculated to be 171° for both **1a** and **1b** is very close to the experimental angle for **1a** (171.0(1)°), but is a poorer estimate for **1b**, which was found experimentally to be 165.8(2)°. The distortion away from the calculated geometry in **1b** is likely caused by packing effects in the solid‐state but at this time it is not possible to conclusively determine whether crystal packing forces are the sole contribution to the distortion.

### Raman Spectroscopy

The Raman spectrum of bulk crystalline [Mn(CO)_5_(*n*‐pentane)]^+^[*alfal*]^−^ (Figure 4b) shows the three expected *ν*(C≡O) vibrations, which match in the position and intensity of the DFT calculated vibrations of **1a** very well. The expected *ν*(C─H) vibrations have not been observed, as they are overlaid by the absorptions of uncoordinated *n*‐pentane present from the 5FB/*n*‐pentane solvent mixture in the flask (Figure 4b, blue line; photo). Yet, we unambiguously observe a broad band at 1601 cm^−1^, which is possibly the *δ*(CH_2_) scissoring vibration of **1a** (calc.: 1568 cm^−1^).

### NMR Characterization

NMR samples of [Mn(CO)_5_(*n*‐pentane)]^+^[*alfal*]^−^, **1**, were prepared by oxidation of Mn_2_(CO)_10_ with 2.2 equivs of the de‐electronator salt [C_10_F_8_]^+·^[*alfal*]^−^ in a solution of HFP at low temperatures (−40 °C) before adding an excess of *n*‐pentane to the reaction mixture. In solution at −90 °C, the high field region of the ^1^H NMR spectrum of [Mn(CO)_5_(*n*‐pentane)]^+^[*alfal*]^−^ shows three broad resonances at δ −3.96, −6.23, and −6.36 with intensities in the ratio approximately 1:2.7:5.9 respectively (Figure 4e) and we assign these signals as the metal‐bound protons in rapid equilibrium with their geminal proton(s). In HFP solution at −90 °C, [Mn(CO)_5_(*n*‐pentane)]^+^, **1**, can be observed for many hours (>7.5 h), enabling detailed characterization by ^1^H NMR spectroscopy. We assign the resonance at δ −3.96, to a metal bound CH_3_, the C1 isomer **1c**. The chemical shift of the resonance for the C1 isomer is substantially upfield of that of the other two isomers, **1a** and **1b**, as the resonance is an average of two noncoordinated hydrogen and one coordinated hydrogen atoms, whereas the resonances at higher field correspond to binding at C2 and C3 where the chemical shifts are the average of one noncoordinated hydrogen‐ and one coordinated hydrogen‐atom. At this time, we cannot assign, which high‐field peak corresponds to the isomers **1a** or **1b**. However, the trend of the predicted chemical shifts δ^1^H agrees with the experiment (**1a**: −6.61; **1b**: −7.19; **1c**: −4.76 ppm; and SI 7.3) and so we tentatively assign the resonances at δ −6.23, −6.36, and −3.96 as **1a**, **1b,** and **1c**, respectively. In either case, whether the assignments of **1a** and **1b** are correct or swapped, the relative energies of **1a** and **1b** are within 2.3 kJ mol^−1^ under these conditions, based upon Boltzmann weighting of the experimental ratios (See the Supporting Information for further calculations of relative energies of isomers, which show **1a** and **1b** to be very close in energy).

Separately, photolysis of a solution of [Mn(CO)_6_]^+^[*alfal*]^−^ and pentane in HFP at −90 °C inside the NMR spectrometer yielded the same 3 broad resonances at δ^1^H −3.96, −6.23, and −6.36, consistent with the data from the oxidative route and corroborating these resonances as the isomers of bound *n*‐pentane. Exchange spectroscopy (EXSY) at −90 °C indicates facile exchange between the three isomers, accounting for the broadness of the resonances ascribed to the metal bound *n*‐pentane. The interchange between isomers **1a–c** is likely a “chain walking” process, where the metal undergoes a series of 1,2‐ or 1,3‐migrations along the pentane chain, binding to the different C─H bonds during the experiment. In the EXSY spectrum, we observed no evidence for exchange between bound and free pentane at −90 °C. Additional resonances that have been assigned as the noncoordinated C─H groups of the bound *n*‐pentane were located by total correlation spectroscopy (TOCSY). However, as the rate of interchange between the isomers of the bound *n*‐pentane is multiple times per second, exchange also occurs during the TOCSY experiment, so it is not possible to assign all of the resonances of the nonbound C─H bond environments to specific isomers. There is no evidence in the ^1^H NMR spectrum for C─H bond cleavage to form the corresponding alkyl hydride complexes, neither in solution, nor the solid state.

### Binding Energies and Bonding Interactions

Since the *n*‐pentane complex, **1**, can be crystallized from solution at room temperature, this relatively high temperature should in principle provide sufficient energy to readily enable competing ligand substitution from the environment, particularly of the very weakly bound *n*‐pentane. Yet, when the Gibbs energies Δ_r_ *G*° of the reaction of the [Mn(CO)_5_]^+^ fragment with the potential ligands *n*‐pentane, 5FB, HFP, and perfluoronaphthalene C_10_F_8_ were calculated at the MN15/def2‐TZVPP level of theory at standard conditions (25 °C, 1 bar), Δ_r_ *G*° toward *n*‐pentane was predicted to be the most favorable (−56 kJ mol^−1^), compared to the alternative binding to 5FB (−36 kJ mol^−1^), HFP (−40 kJ mol^−1^), or C_10_F_8_ (−40 kJ mol^−1^). Even the least favorable C1‐bound isomer **1c** binds with an energy of −45 kJ mol^−1^ and, hence, binds more strongly than any of the solvent/substrate competitors. Our experimental and theoretical results clearly illustrate that the system which we have developed, promotes alkane binding as a “regular ligand”, simply because this is the strongest ligand available in solution for the metal complex.

To compare the M─H interactions of the [Mn(CO)_5_(*n*‐pentane)]^+^ complex with the neutral isoelectronic Cr(CO)_5_(alkane) complexes first investigated in the 1970′s by Kelly and Perutz et al.,^[^ ^50^, ^51^, ^52^, ^53^, ^54^, ^55^, ^56^, ^57^, ^58^ ^]^ we performed an atoms in molecules (AIM) analysis at the MN15/def2‐TZVPP level for **1a−c** and the C3 bound isomer of Cr(CO)_5_(*n*‐pentane) (Figure 4c). The calculated electron densities residing on the bond critical points (BCPs) for Mn─H bonds (0.32−0.34 *e* ^−^ Å^−3^) are significantly higher than those in the Cr─H bond (0.24 *e* ^−^ Å^−3^). By contrast, the electron densities are most comparable to the alkane *σ*‐complexes reported by Weller et al.^[^ ^12^ ^]^ Despite the Weller systems having a different electron count and ligand scaffold, the BCPs of these cationic, group nine species range from *ca*. 0.32 *e* ^−^ Å^−3^ to ca. 0.40 *e* ^−^ Å^−3^, i.e., very similar to that observed for **1a–c**. In addition, the coordination of the alkane yields to a drastic weakening of the C─H bond BCPs from ca. 1.9 to 1.5 *e* ^−^ Å^−3^ in all isomers.

Energy decomposition analyses with natural orbitals of chemical valence (EDA‐NOCV; Figure 4d) yielded the orbital interactions Δ*E*_orb_ between *n*‐pentane and [Mn(CO)_5_]^+^ in the range between 114 and 124 kJ mol^−1^ in **1a–c**. They are mainly composed (>70 % of Δ*E*_orb_) of the *σ*‐donations of the C─H centered HOMO of *n*‐pentane into the *d*_z_2‐centred LUMO of [Mn(CO)_5_]^+^, with only minimal (*ca*. 10 % of Δ*E*_orb_) *π*‐back donations into the σ*(C─H) orbital in all the isomers **1a–c**. Dispersion interactions, Δ*E*_disp_, further stabilize **1a–c** by 38 to 47 kJ mol^−1^.

## Conclusions

Here we report [Mn(CO)_5_(*n*‐pentane)]^+^ as a long‐lived metal alkane complex formed in solution by directly binding pentane as an incoming ligand to [Mn(CO)_5_]^+^. The complex [Mn(CO)_5_(*n*‐pentane)]^+^ is stable in the solid state at room temperature and was characterized by single crystal x‐ray diffraction, Raman and solution state NMR spectroscopic techniques. In both, the solid and the solution state, [Mn(CO)_5_(*n*‐pentane)]^+^ exhibits a binding preference for the C2 and C3 bound isomers **1b** and **1a** respectively over the C1 isomer **1c** and we see no evidence for C─H bond cleavage to form the alkyl hydride species. This is the first report of both solid and solution state analysis of a σ‐alkane complex and this report is the only example of an alkane *σ*‐complex crystallized from the solution state. In synthesizing [Mn(CO)_5_(*n*‐pentane)]^+^ we have developed a new method for accessing highly reactive complexes by generating a reactive metal cation by chemical oxidation of a metal precursor in the presence of an alkane substrate in an inert solvent and in the presence of a weakly coordinating anion. The synthetic route provides a robust synthetic strategy for the study of metal interactions with other weakly binding substrates. Our system is a significant step forward in the field of alkane activation and more widely in the field of reactive organometallic chemistry. While, in this report, we have examined only the reactive [Mn(CO)_5_]^+^ species, it is clear that the method can be extended to the study of a range of other metal complexes, binding other alkanes and organic substrates as well as other nonorganic substrates.

## Author Contributions

Conceptualization: M.S., I.K. Methodology: M.S., J.D.W., G.E.B., L.D.F., and I.K. Investigation: M.S., J.D.W., J.F., and G.E.B. Visualization: M.S., J.D.W. Funding acquisition: G.E.B., L.D.F., and I.K. Project administration: G.E.B., L.D.F., and I.K. Supervision: G.E.B., L.D.F., and I.K. Writing — original draft: M.S., J.D.W. Writing — review & editing: M.S., J.D.W., G.E.B., L.D.F., and I.K.

## Conflict of Interests

The authors declare no conflict of interest.

## Supporting information



Supporting information

Supporting information

## Data Availability

The data that support the findings of this study are available in the Supporting Information of this article. A preliminary version of this manuscript has been deposited on the preprint server ChemRxiv: https://doi.org/10.26434/chemrxiv‐2025‐ffpdw Deposition Numbers 2416533 (for [Mn(CO)_5_(*n*‐pentane)]^+^[*pf*]^−^) and 2416534 (for [Mn(CO)_5_(*n*‐pentane)]^+^[*alfal*]^−^) accessible at https://www.ccdc.cam.ac.uk/services/structures?id=10.1002/anie.202507494, contain the supplementary crystallographic data for this paper. These data are provided free of charge by the joint Cambridge Crystallographic Data Centre and Fachinformationszentrum Karlsruhe http://www.ccdc.cam.ac.uk/structures.
