# Supplementary material for: Promoting Alkane Binding: Crystallization of a Cationic Manganese(I)‐Pentane σ‐Complex from Solution
Source: Angew Chem Int Ed Engl. 2025 May 27;64(27):e202507494. doi: 10.1002/anie.202507494 (PMC12207380; doi:10.1002/anie.202507494)
Supplement: Supplementary file 1 — Supporting information [file ANIE-64-e202507494-s002.docx]

**Supplementary Information**

**Promoting Alkane Binding: Crystallization of a Cationic Manganese(I)‑Pentane *σ*-Complex from Solution**

**Authors:** Malte Sellin,^1^† James D. Watson,^2^† Julia Fischer,^1^ Graham E. Ball,^2^ Leslie D. Field,^2^ Ingo Krossing^1^*

**Affiliations:**

^1^ Institut für Anorganische und Analytische Chemie and Freiburg Materials Research Center FMF, University of Freiburg, 79104 Freiburg, Germany

^2^ School of Chemistry, UNSW Sydney, NSW 2052, Australia

* Corresponding author. Email: ingo.krossing@ac.uni-freiburg.de

† These authors contributed equally to this work

**This file includes:**

**Methods**

**Supplementary Text**

**Figures S1 to S16**

**Tables S1 to S10**

Contents

[1. Compounds, compound numbering and abbreviations 3](#_Toc196175985)

[2. General Procedures 4](#_Toc196175986)

[3. Synthetic Procedures 6](#_Toc196175987)

[4. Single Crystal X-Ray Diffraction 8](#_Toc196175988)

[5. Raman Spectroscopy 12](#_Toc196175989)

[6. Supplementary NMR Spectra 14](#_Toc196175990)

[7. DFT Calculations 19](#_Toc196175991)

[7.1 EDA-NOCV Results 19](#_Toc196175992)

[7.2 AIM Analysis 20](#_Toc196175993)

[7.3 NMR Calculations 24](#_Toc196175994)

[7.4 B3LYP Optimized Structures 26](#_Toc196175995)

[7.5 MN15 Optimized Structures 29](#_Toc196175996)

[7.6 Calculations of relative energies of conformations of [Mn(CO)_5_(*n*- pentane)]^+^ (**1a-c**) 35](#_Toc196175997)

[8 References 36](#_Toc196175998)

# Compounds, compound numbering and abbreviations

# General Procedures

All manipulations were carried out by using standard Schlenk technique or a nitrogen filled glovebox (O_2_/H_2_O < 0.1 ppm). All the reactions were performed in Schlenk tubes with grease free PTFE-valves. The solvent 1,2,3,4-tetrafluorobenzene (4FB, C_6_F_4_H_2_, from Fluorochem) was stirred a few days over calcium hydride (CaH_2_) and distilled. The distillate was stirred over Ag^+^[Al(OR^F^)_4_]^−^ (R^F^ = C(CF_3_)_3_) and condensed to remove traces of less fluorinated benzenes. This leads to a minor contamination of R^F^OH (<1 %), which does not affect the reactions. The solvent pentafluorobenzene (5FB) was stirred over calcium hydride (CaH_2_) for 48 hours and distilled. 1,1,1,3,3,3–Hexafluoropropane (HFP) was purified in 20 mL portions by stirring over ~2 g of Ag^+^[Al(OR^F^)_4_]^−^ and ~200 mg of Mn_2_(CO)_10_ at −40 °C before the volatiles were transferred and collected *via* liquid to liquid vacuum distillation at −20 °C to remove traces of less fluorinated alkanes and the persistent chlorotrifluoroethane impurity from the HFP. *n*-Pentane was dried using a Grubbs apparatus. 4FB, 5FB, HFP and *n*‑pentane were stored over 3 Å molar sieves. Octafluoronaphthalene (ABCR) and Mn_2_(CO)_10_ (ChemPur) were bought from commercial sources and used as received. [C_10_F_8_]^+∙^[F{Al(OR^F^)_3_}_2_]^− [1]^ and Ag^+^[Al(OR^F^)_4_]^− [2]^ were prepared according to literature procedures.

**Raman Spectroscopy**

FT Raman spectra were recorded with a VERTEX 70 with Bruker RAM II Modul (1064 nm exciting line of a Nd-YAG laser) and liquid nitrogen cooled Ge detector. The samples were measured at RT in the range of 4000−30 cm^–1^ with 4,000 scans and a resolution of 4 cm^–1^ with a laser power of 20 mW. The intensities are reported as follows: ≥ 0.8 = very strong (vs), ≥ 0.6 = strong (s), ≥ 0.4 = medium (m), ≥ 0.2 = weak (w), < 0.2 = very weak (vw). The data were processed with the Bruker OPUS 7.5 software package. The graphical representations were created with OriginPro 2021.

**NMR Spectroscopy**

NMR spectra were recorded at −90 °C on a Bruker Avance III 700 NMR spectrometer fitted with a TXI probe and operating at 700 MHz. The NMR spectra are referenced against tetramethylsilane.

Acquisition of NMR Data for Alkane *σ*‑Complexes

The NMR experiments that were used to analyze the alkane *σ*‑complexes were performed at low temperatures in protio HFP. HFP does not have any deuterium nuclei and therefore all NMR spectra were collected without a deuterium lock and shimmed by optimizing signals from the ^1^H nuclei. To achieve a starting point for shimming on HFP containing samples, the spectrometer was first shimmed (at the experimental temperature, typically around −90 °C) on a sample of acetone in *d*_6_ acetone (20:80) containing a single drop of CH_2_Cl_2_ to provide a sharp resonance to use as a reference whilst performing manual shimming. Once the shimming on the acetone-*d_6_*:acetone:DCM sample was acceptable (peak width at half height of the singlet DCM resonance at δ 5.63 is < 3 Hz), the lock and sweep were turned off, the acetone-*d_6_*:acetone:DCM sample was ejected, and the sample for analysis was lowered into the NMR spectrometer. Standard Bruker pulse sequences were deployed for solvent suppression (zgesgp); TOCSY experiments (mlevphpro1); and nOe experiments with solvent suppression (noesygpphprrso1).

Preparation of NMR Samples

***Precautionary safety note: 1,1,1,3,3,3-Hexafluoropropane (HFP) boils at ca. 272 K: Samples must be kept at temperatures well below 270 K to mitigate the risk of explosion by the build-up of pressure if CF_3_CH_2_CF_3_ is used above its boiling point in a sealed vessel.***

Sample Preparation for *in situ* Deelectronation Experiments

Typically, a pre-dried J. Young NMR tube and standard J. Young NMR tube piston, were cycled into an argon filled glove box. 1 mg of dimanganese decacarbonyl and 15 mg (2.0 eq.) of the deelectronator salt, [C_10_F_8_]^+∙^[*alfal*]^−^ were charged into the bottom of the tube before the tube was sealed using the J. Young NMR tube piston. Samples were cycled out of the glove box before being dried *in vacuo* to remove any volatile contaminants. After the complex had been exposed to a vacuum, HFP (600-700 μL) was transferred onto the reagents *via* trap-to-trap vacuum distillation. Samples were mixed using a vortex at mixer −40 °C until homogenous. While mixing, the samples typically changed color from dark green to pale green/yellow. Once homogeneous, the samples were frozen once more and dry, degassed *n*‑pentane (10-15 μL) was distilled onto the J. Young NMR tube *via* trap-to-trap vacuum distillation. Once the *n*‑pentane was added to the reaction mixture, the samples were mixed briefly before being stored in an ethanol cold bath at temperatures below −80 °C and transported to the NMR spectrometer ready for analysis. Samples were removed from the cold bath and wiped several times with dry tissue paper to remove excess ethanol prior to inserting into a precooled and shimmed NMR spectrometer.

Sample Preparation for *in-situ* Photolysis

NMR samples for photolysis were prepared in J. Young NMR tubes that could be fitted with a modified, bespoke J. Young NMR tube cap (apparatus assembly described previously)^[3]^ with an optical fiber coupling machined from Polyether Ether Ketone (PEEK).

Typically, a modified J. Young NMR tube cap and a pre-dried J. Young NMR tube, were cycled in to an argon or a nitrogen filled glove box where 1−2 mg of the desired tricarbonyl precursor complex, [Mn(CO)_6_]^+^[WCA]^−^ was charged into the NMR tube before the tube was sealed using the modified J. Young NMR tube cap. Samples were cycled out of the glove box before being dried *in vacuo* for at least 24 h prior to photolysis in order to remove any volatile contaminants. After the complex had been exposed to a vacuum for an extended period, the desired HFP/alkane solvent mixtures were transferred onto the complex via trap-to-trap vacuum distillation. Samples were mixed using a vortex mixer until homogenous at low temperatures below 0 °C.

Samples were connected to the PEEK NMR tube and optical fiber couplers, coupled to an optical fiber connected to a 100 W Hg arc lamp and inserted into a precooled and shimmed NMR spectrometer using the optical fiber as a tether. Once the sample temperature had settled (~10 minutes) the sample was shimmed manually until the lineshape and peak widths at half-height were acceptable (Δν_1/2_ = < 3 Hz) before the UV lamp was turned on and the sample was irradiated.

Preparation of Alkane/HFP Solvent Mixtures with Liquid Alkanes for photolysis

Mixtures of HFP and liquid alkanes were prepared as needed. *n*‑Pentane (10-15 μL) was measured into a standard J. Young NMR tube in a nitrogen or argon filled glove box using a micro syringe. The alkane was degassed (using three freeze-pump-thaw cycles). In a separate, pre-dried, standard J. Young NMR tube, HFP (600-700 μL) was vacuum transferred via trap-to-trap distillation and then degassed (by three freeze-pump-thaw cycles). The degassed alkane was transferred via trap-to-trap vacuum distillation into the J. Young NMR tube containing the HFP. The solution was allowed to thaw (whilst keeping the sample cold) before it was mixed on a vortex mixer. It should be noted that > ~5 % v/v HFP/pentane will phase separate when the solution is cooled to low temperatures (< ‑80 ℃). This results in droplets of alkane forming and produces a second set of alkane resonances in the ^1^H and ^13^C NMR spectra. This does not result in a decrease in the yield of photolysis products but can interfere with the magnetic field homogeneity and can make shimming more challenging and obscure some resonances in the ^1^H NMR spectrum.

Single Crystal X-ray Diffraction

The data were collected on a Bruker D8 VENTURE dual wavelength Mo/Cu three-circle diffractometer with a microfocus sealed X-ray tube using mirror optics as monochromator and a Bruker PHOTON III detector. Single crystals were selected at RT in PFPE oil JC 1800 (Sunoit Performance Material Science), mounted on CryoLoops with a diameter of 0.1 to 0.2 mm and shock-cooled using an Oxford Cryostream 800 low temperature device. The data were gathered at 100(2) K using Mo K_α_ radiation (λ = 0.71073 Å). All data were integrated with SAINT (version 8.38A) and a multi-scan absorption correction using SADABS or TWINABS was applied. The structures were solved by direct methods using SHELXT^[3]^ and refined by full-matrix least-squares methods against F^2^ by SHELXL-2018/3^[4]^ using the GUI software ShelXle.^[5]^ Disordered moieties were refined using bond lengths restraints and displacement parameter restraints and were modelled with the program DSR.^[6]^ The gathered data were finalized with the tool FinalCif.^[8]^ The graphical representations of the crystal structures were generated with Mercury (version 4.0).^[8]^ Crystallographic data for the structures reported in this paper have been deposited with the Cambridge Crystallographic Data Centre.^[9]^ Copies of the data can be obtained free of charge from the Cambridge Crystallographic Data Centre via www.ccdc.cam.ac.uk/structures.

Computational Details

Geometry optimizations were performed with the TURBOMOLE software^[11]^ (v7.2 or v7.5) using the DFT functionals B3LYP^[12]^ or MN15^[13]^ with the def2-TZVPP^[13]^ basis set, the resolution-of-identity (RI) approximation,^[14]^ dispersion correction (D3BJ, only for B3LYP),^[15]^ a fine integration grid (m4 or 5a for NMR chemical shift calculations) and the default SCF convergence criteria (10^–6^ a.u.). All structures were checked for proper spin occupancies and imaginary frequencies with the integrated *EIGER* and *AOFORCE*^[16]^ modules. The Raman spectrum was simulated at B3LYP(D3BJ)/def2-TZVPP level with a scaling factor of 0.968.^[18]^

# Synthetic Procedures

[Mn(CO)_5_(*n*-pentane)]^+^[*alfal*]^−^ **(1)**

[C_10_F_8_]^+∙^[*alfal*]^−^ (71 mg, 35 μmol, 2.0 eq.) was dissolved in 5FB (1 mL) in a Schlenk tube and cooled to −20 °C. Mn_2_(CO)_10_ (4.5 mg, 18 μmol, 1.0 eq.) was dissolved in 5FB (1mL) in a second Schlenk tube and also cooled to −20 °C. The solution containing the Mn_2_(CO)_10_ was added slowly to the first solution. During the addition and subsequent stirring for 1 hour at −20 °C, the colour of the reaction solution changed from green to red/orange. Finally, *n*‑pentane (10 mL) was layered on top of the solution. Slow diffusion of the two solvents at 0 °C over days afforded yellow/orange crystals of [Mn(CO)_5_(*n*‑pentane)]^+^[*alfal*]^−^. The Raman spectrum of this compound was measured directly in the Schlenk tube after the removal of the solvent mixture (Figure S1). Caution! Full removal of the solvent leads to the decomposition of the alkane complex. No yield was determined.

Raman ν̃ [cm^-1^] = 3094 (vw), 2964 (m), 2936 (s), 2915 (s), 2902 (s), 2876 (vs), 2734 (vw), 2302 (vw), 2177 (vw), 2166 (w), 2134 (vw), 2123 (w), 2074 (vw), 1601 (vw), 1459 (vw), 1444 (vw), 1299 (vw), 1284 (vw), 1144 (vw), 1074 (vw), 1029 (vw), 994 (vw), 955 (vw), 906 (vw), 868 (vw), 840 (vw), 815 (vw), 766 (vw), 753 (w), 719 (vw), 578 (vw), 540 (vw), 470 (vw), 470 (vw), 437 (vw), 401 (w), 367 (vw), 326 (vw), 293 (w), 232 (vw), 166 (vw), 110 (vs), 85 (m).


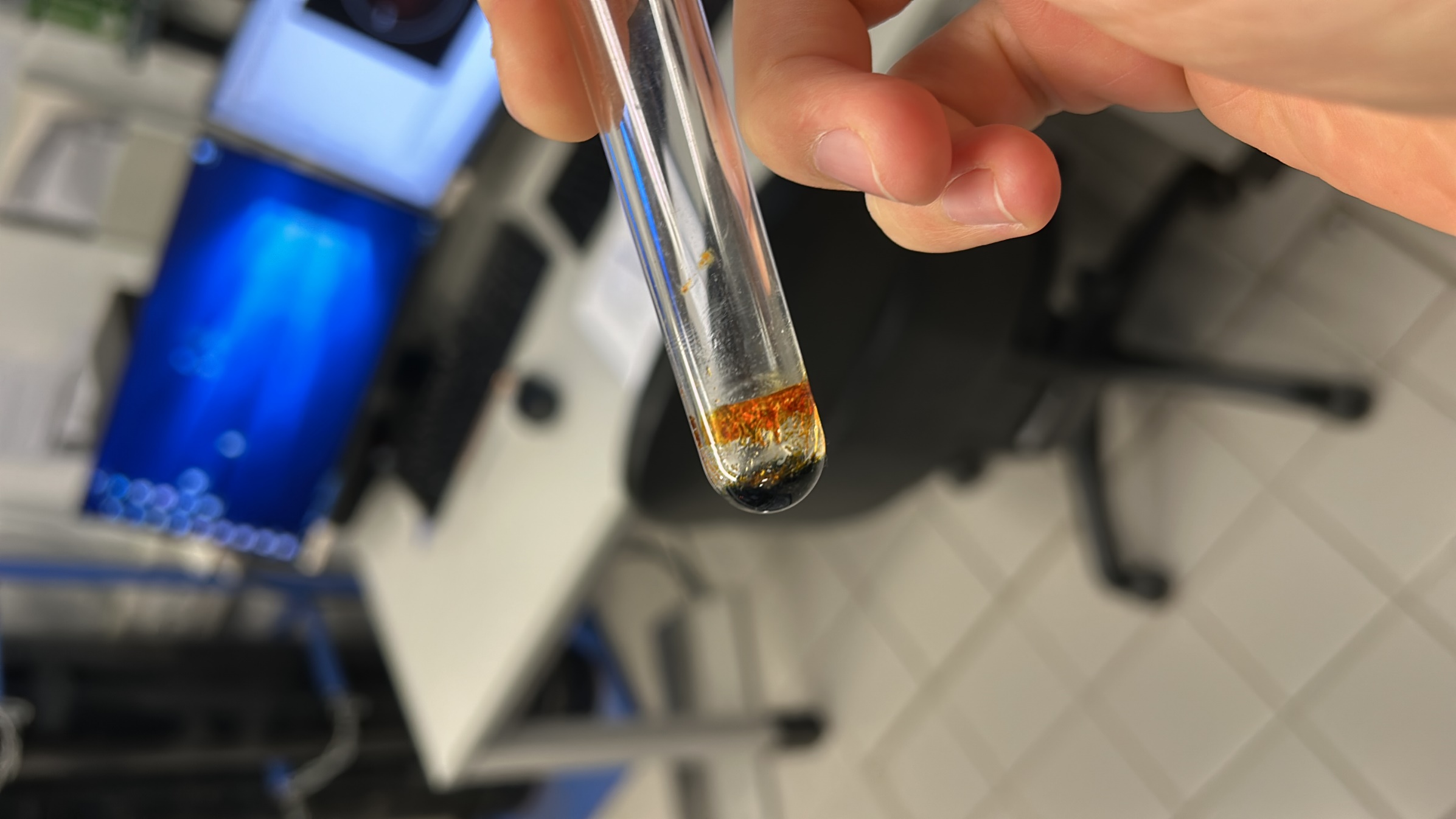


Figure S1: Schlenk tube after the removal of the solvent mixture. The orange ring of single crystals is [Mn(CO)_5_(*n*‑pentane)]^+^[*alfal*]^−^ and the green to black powder at the bottom of the tube is excess [C_10_F_8_]^+∙^[*alfal*]^−^.

[Mn(CO)_5_(*n*-pentane)]^+^[*pf*]^−^

Ag^+^[*pf*]^−^ (50 mg, 47 μmol, 2.0 eq.) and Mn_2_(CO)_10_ (9.0 mg, 23 μmol, 1.0 eq.) were dissolved in 4FB (1 mL) and stirred for one hour at −20 °C. The reaction solution was filtered and the filtrate was layered with *n*‑pentane (10 mL). Slow diffusion of the two solvents at temperatures from −20 °C to +25 °C over days yielded a mixture of single-crystalline [Mn(CO)_6_]^+^[*pf*]^−^, [Mn(CO)_5_(4FB)]^+^[*pf*]^−^ and [Mn(CO)_5_(*n*‑pentane)]^+^[*pf*]^−^ besides black precipitate (Figure S2).


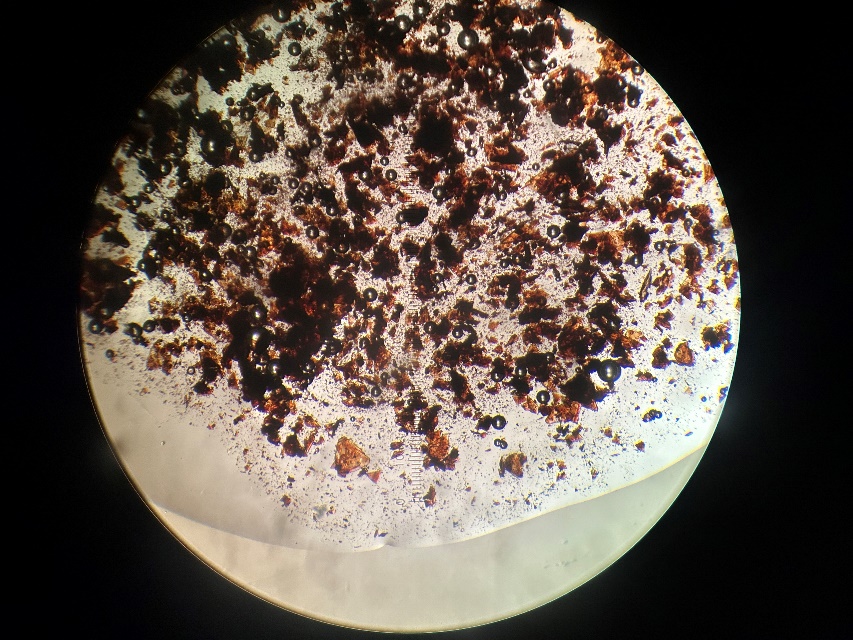


Figure S2: Mixture of several crystalline species with black amorphous precipitate.

Despite many attempts, this procedure could not be further optimized. Slight changes of this procedure yielded different results. Exchange of the solvent from 4FB to 5FB only yielded microcrystalline powders. Exchange of the anion from [*pf*]^−^ to [*alfal*]^−^ exclusively yielded [Mn(CO)_5_(4FB)]^+^[*alfal*]^−^ as the product.

# Single Crystal X-Ray Diffraction

Table S1: scXRD data of [Mn(CO)_5_(*n*-pentane)]^+^[*alfal*]^−^ and [Mn(CO)_5_(*n*-pentane)]^+^[*pf*]^−^.

| Compound | [Mn(CO)_5_(*n*-pentane)]^+^[*alfal*]^−^ | [Mn(CO)_5_(*n*-pentane)]^+^[*pf*]^−^ |
| --- | --- | --- |
| CCDC number | 2416534 | 2416533 |
| Empirical formula | C_34_H_12_Al_2_F_55_MnO_11_ | C_26_H_12_AlF_36_MnO_9_ |
| Formula weight | 1750.34 | 1234.28 |
| Temperature [K] | 100(2) | 100(2) |
| Crystal system | Triclinic | orthorhombic |
| Space group (number) | $P\overline{1}$ (2) | $P2_{1}2_{1}2_{1}$ (19) |
| *a* [Å] | 16.282(7) | 9.869(4) |
| *b* [Å] | 16.486(10) | 14.625(3) |
| *c* [Å] | 22.177(18) | 28.136(5) |
| α [°] | 75.13(2) | 90 |
| β [°] | 75.633(17) | 90 |
| γ [°] | 85.425(13) | 90 |
| Volume [Å^3^] | 5573(6) | 4061(2) |
| *Z* | 4 | 4 |
| *ρ*_calc_ [gcm^−3^] | 2.086 | 2.019 |
| *μ* [mm^−1^] | 0.507 | 0.562 |
| *F*(000) | 3400 | 2408 |
| Crystal size [mm^3^] | 0.162×0.209×0.334 | 0.193×0.205×0.220 |
| Crystal colour | Orange | yellow to orange |
| Crystal shape | Block | block |
| Radiation | Mo*K_α_* (λ=0.71073 Å) | Mo*K_α_* (λ=0.71073 Å) |
| 2θ range [°] | 2.56 to 53.11 (0.79 Å) | 2.89 to 66.56 (0.65 Å) |
| Index ranges | −20 ≤ h ≤ 20 −20 ≤ k ≤ 20 −27 ≤ l ≤ 27 | −15 ≤ h ≤ 12 −20 ≤ k ≤ 22 −42 ≤ l ≤ 41 |
| Reflections collected | 159682 | 82175 |
| Independent reflections | 22946  *R*_int_ = 0.1200 *R*_sigma_ = 0.0707 | 13906  *R*_int_ = 0.0749 *R*_sigma_ = 0.0434 |
| Completeness to  θ = 25.242° | 99.7 % | 99.8 % |
| Data / Restraints / Parameters | 22946 / 85535 / 3442 | 13906 / 6620 / 938 |
| Absorption correction T_min_/T_max_ (method) | 0.6845 / 0.7454  (multi-scan) | 0.6569 / 0.7465  (multi-scan) |
| Goodness-of-fit on *F*^2^ | 1.048 | 1.056 |
| Final *R* indexes  [*I*≥2σ(*I*)] | *R*_1_ = 0.0871 w*R*_2_ = 0.2239 | *R*_1_ = 0.0392 w*R*_2_ = 0.0955 |
| Final *R* indexes  [all data] | *R*_1_ = 0.1213 w*R*_2_ = 0.2493 | *R*_1_ = 0.0556 w*R*_2_ = 0.1025 |
| Largest peak/hole [eÅ^−3^] | 0.98/−1.89 | 0.44/−0.64 |


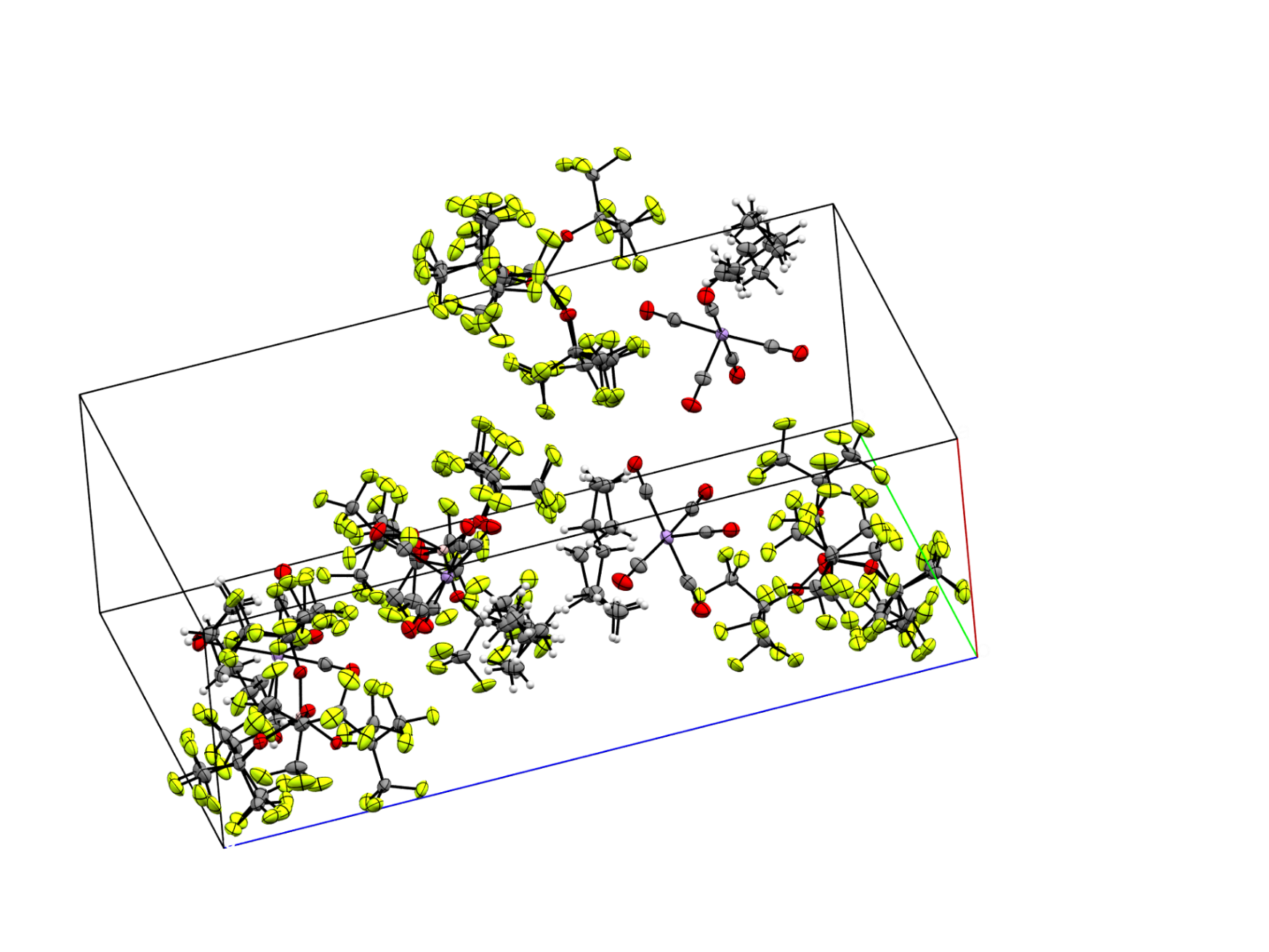


Figure S3: Unit cell of [Mn(CO)_5_(*n*‑pentane)]^+^[*pf*]^−^ with anion and alkane disorders. Displacement ellipsoids shown at 50 % probability level. Color code: manganese – lavender, aluminium – rose, fluorine – light green, oxygen – red, carbon – gray, hydrogen – white.


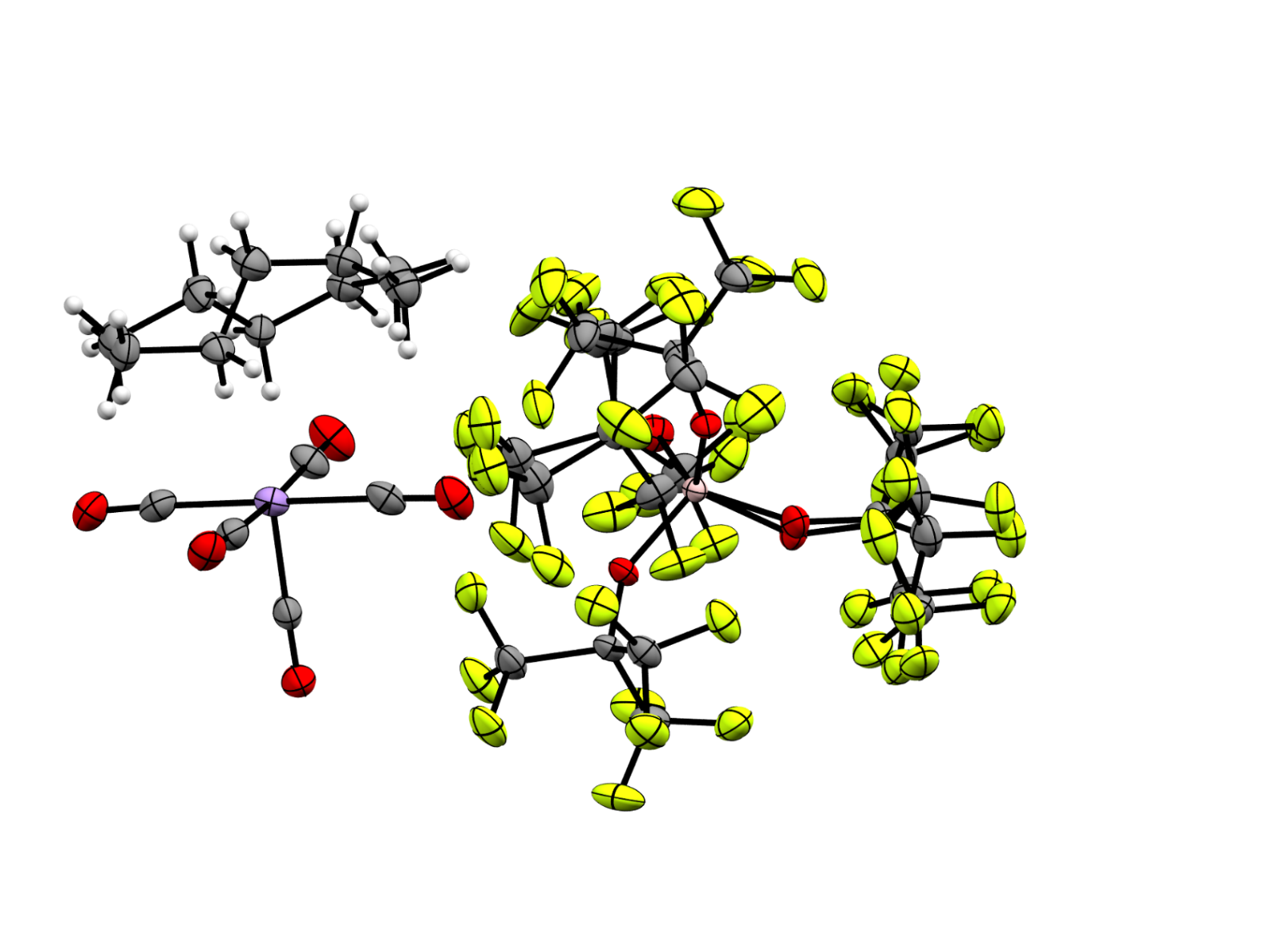


Figure S4: Asymmetric unit of [Mn(CO)_5_(*n*‑pentane)]^+^[*pf*]^−^ with anion and alkane disorders. Displacement ellipsoids shown at 50 % probability level. Color code: manganese – lavender, aluminium – rose, fluorine – light green, oxygen – red, carbon – gray, hydrogen – white.


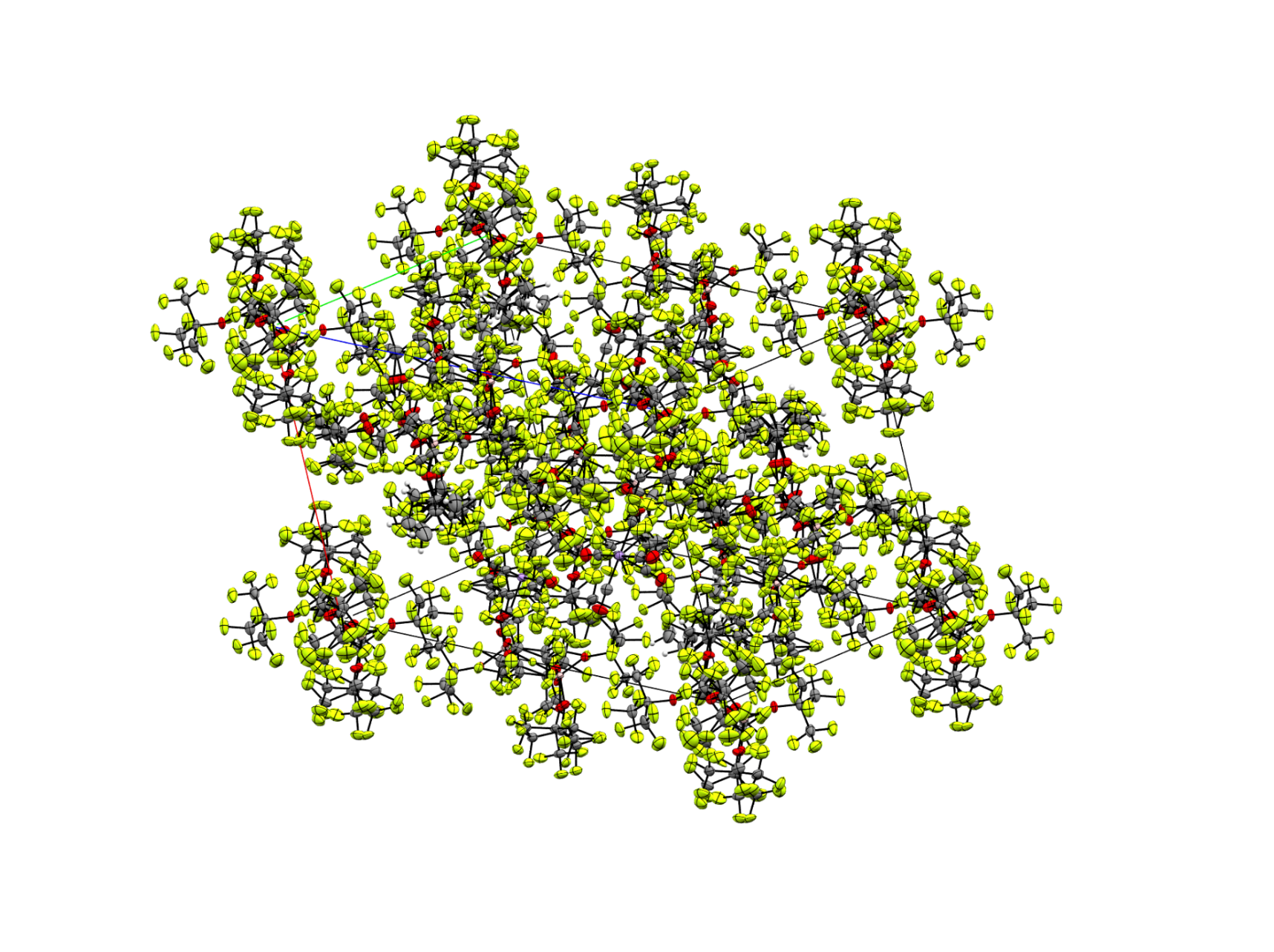


Figure S5: Unit cell of [Mn(CO)_5_(*n*‑pentane)]^+^[*alfal*]^−^ with anion and alkane disorders. Displacement ellipsoids shown at 50 % probability level. Color code: manganese – lavender, aluminium – rose, fluorine – light green, oxygen – red, carbon – gray, hydrogen – white.


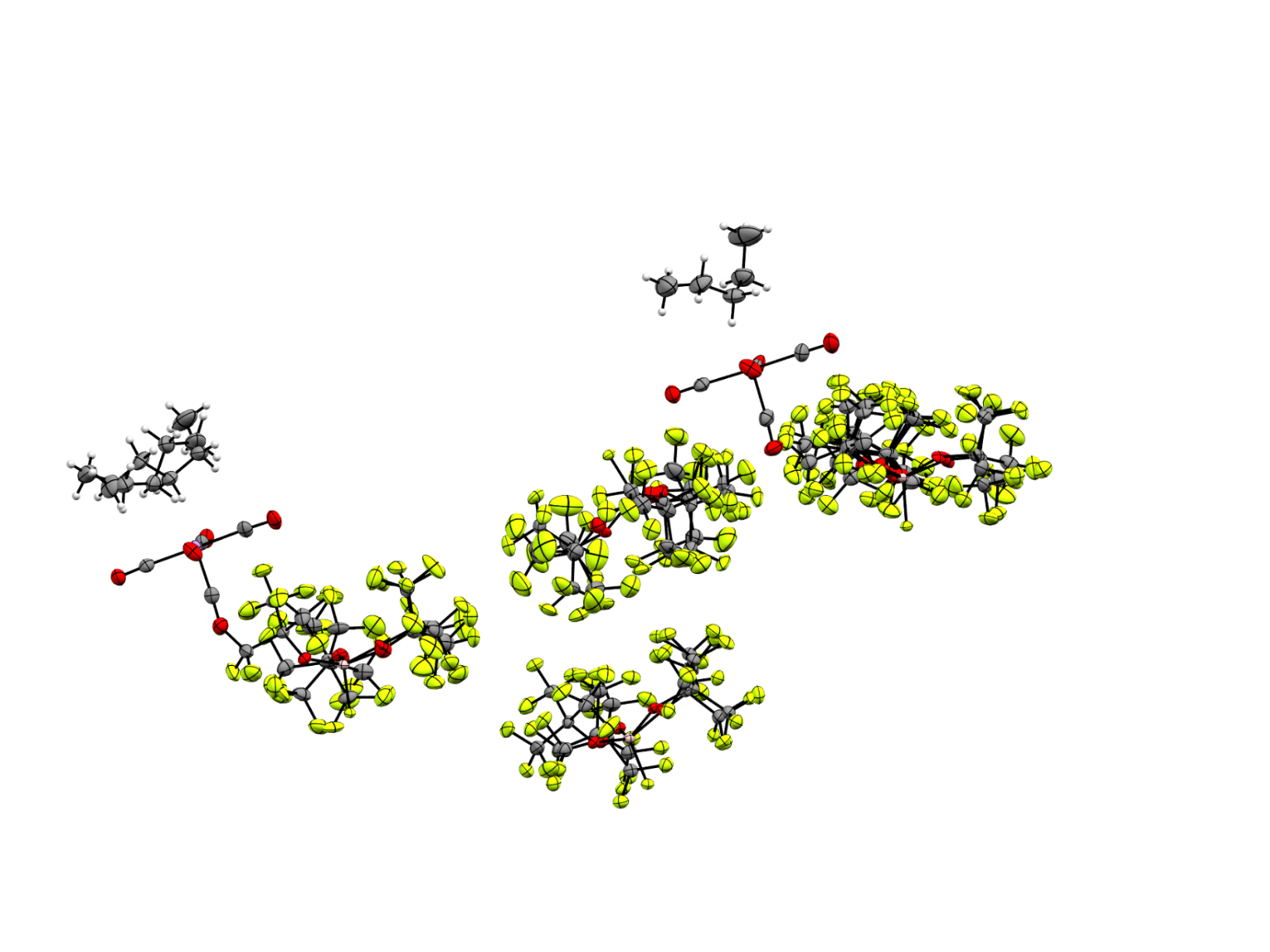


Figure S6: Asymmetric unit of [Mn(CO)_5_(*n*‑pentane)]^+^[*alfal*]^−^ with anion and alkane disorders. Displacement ellipsoids shown at 50 % probability level. Color code: manganese – lavender, aluminium – rose, fluorine – light green, oxygen – red, carbon – gray, hydrogen – white.


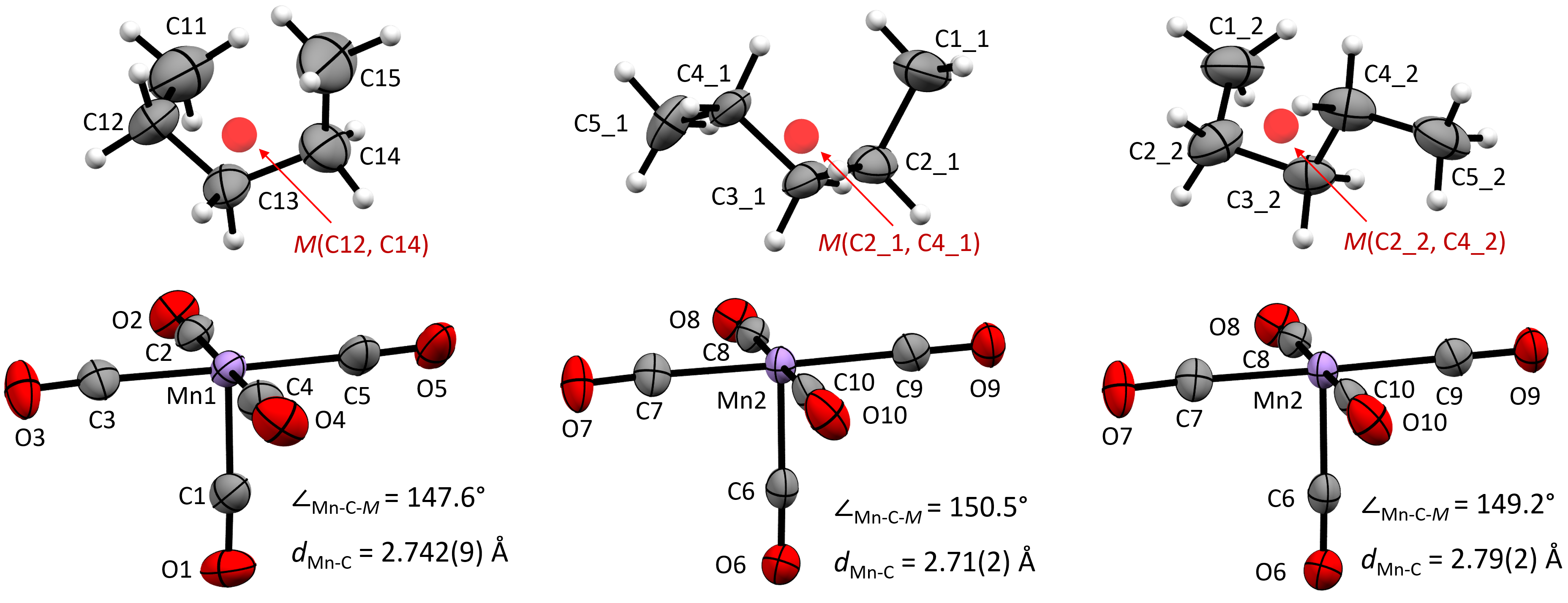


Figure S7: The [Mn(CO)_5_(*n*-pentane)]^+^ moieties of [Mn(CO)_5_(*n*‑pentane)]^+^[*alfal*]^−^. Displacement ellipsoids shown at 50 % probability level. Note that in all cases, the **1a** isomer is observed, only in different conformers. Additionally, the midpoints of the carbon atoms neighboring the coordinating carbon atom are shown (red spheres), which aids the determination of the coordination mode (see manuscript).

# Raman Spectroscopy

Figure S 8: Raman spectrum of [Mn(CO)_5_(*n*-pentane)]^+^[*alfal*]^−^ (black line) with applied baseline correction (red line).


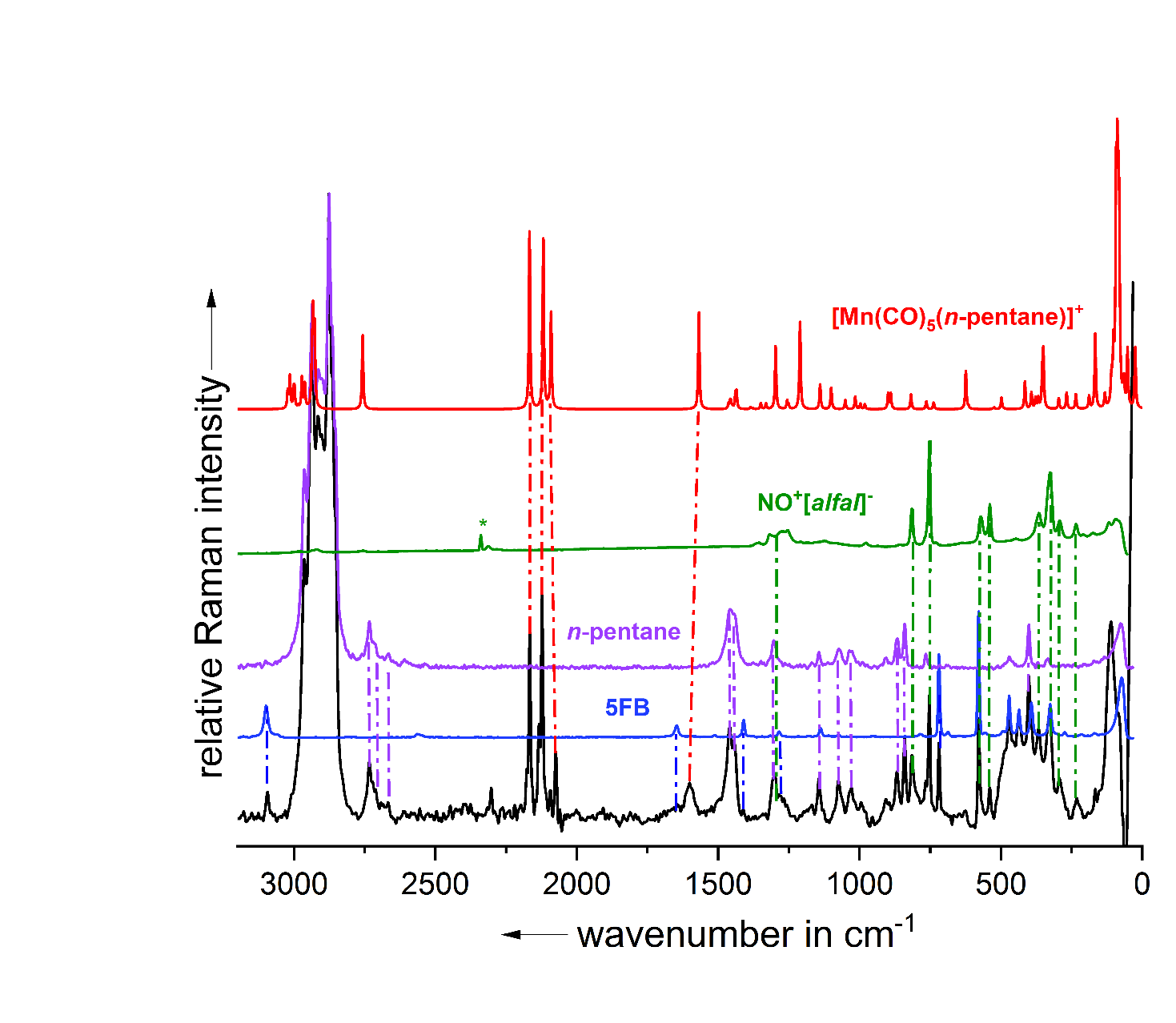


Figure S9: Baseline corrected Raman spectrum of [Mn(CO)_5_(*n*-pentane)]^+^[*alfal*]^−^ (black line) in comparison to the Raman spectra of 5FB (blue line), *n*-pentane (lavender line), NO^+^[*alfal*]^−^ (residual from synthesis of [C_10_F_8_]^+∙^[*alfal*]^−^ and as reference for the [*alfal*]^−^ anion; green line) and the B3LYP(D3BJ)/def2-TZVPP calculated spectrum of [Mn(CO)_5_(*n*-pentane)]^+^ (red line) scaled by 0.968 according to Duncan *et al*.^[18]^ (*) denotes the vibration from the nitrosonium cation.


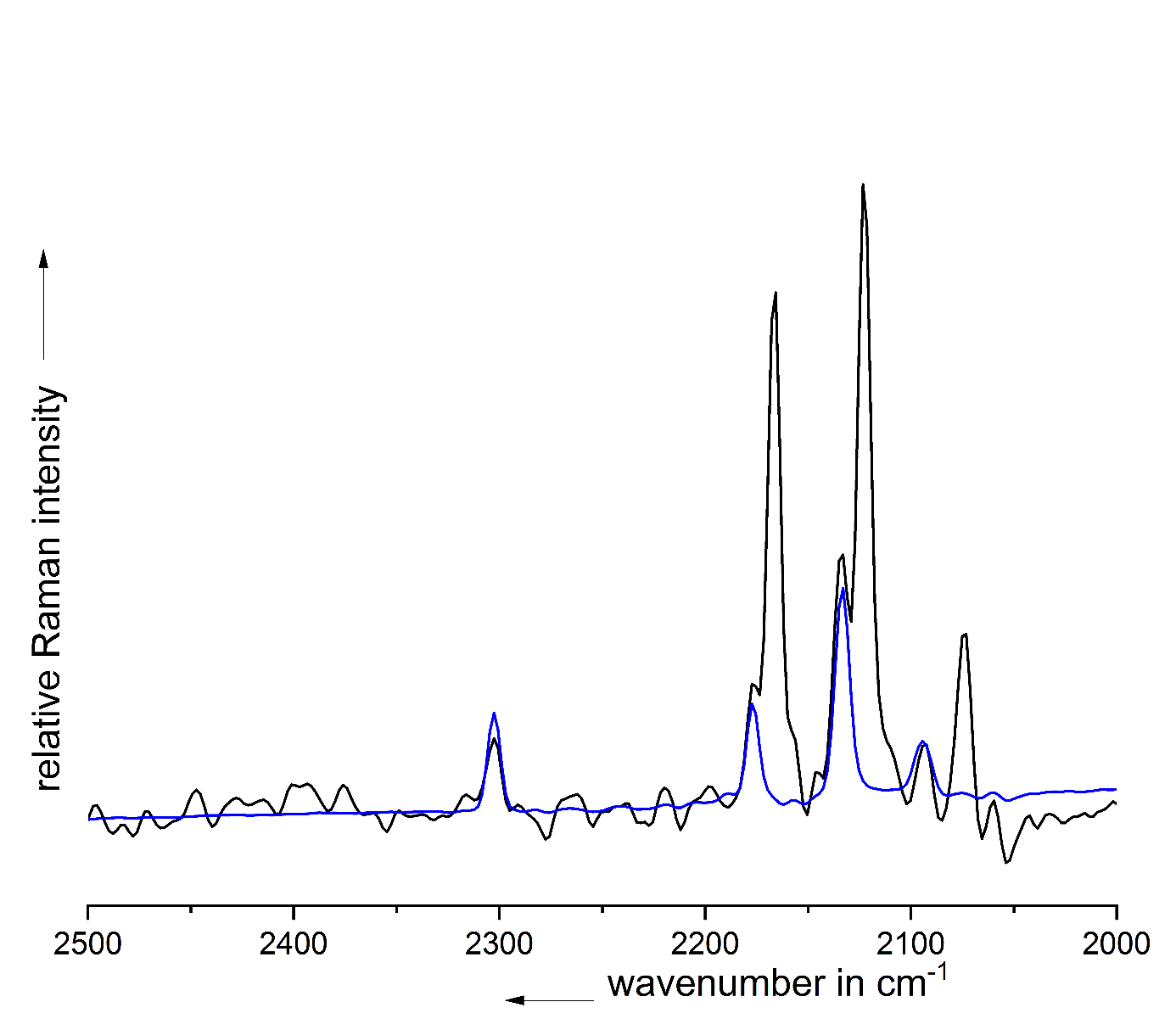


Figure S10: Baseline corrected Raman spectrum of [Mn(CO)_5_(*n*-pentane)]^+^[*alfal*]^−^ (black line) in comparison to the Raman spectrum of the reaction product, when the reaction is carried out under a dinitrogen atmosphere (blue line).

Figure S11: Raman spectrum of the product (black line), when the reaction is carried out under a dinitrogen atmosphere in comparison with the B3LYP(D3BJ)/def2-TZVPP calculated Raman spectrum of [Mn(CO)_5_(N_2_)]^+^ (red line) scaled by 0.968 according to Duncan *et al.*^[18]^ As this complex is intended to be published elsewhere, it is not discussed further here.

# Supplementary NMR Spectra


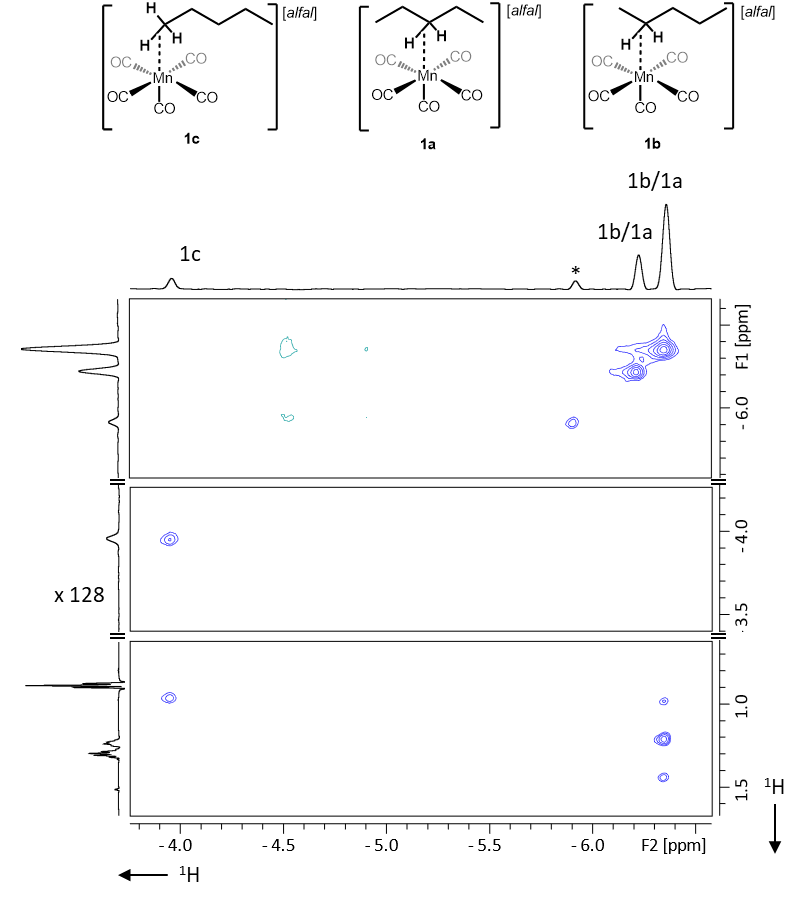


Figure S12 The high field region of a 700 MHz 2D ^1^H-^1^H TOCSY NMR spectrum of the three isomers of [Mn(CO)_5_(*n*‑pentane)]^+^ (**1a-c**) generated by oxidation of Mn_2_(CO)_10_ by [C_10_F_8_]^+∙^[*alfal*]^−^ in the presence of *n*-pentane, collected at −90 °C in HFP solvent with a TOCSY mixing time of 10 ms.*denotes a the complex [Mn(CO)_5_(*c*‑pentane)]^+^ formed as a result of contamination of *n*-pentane with *c*-pentane (*ca* 4% mol/mol). Some cross peaks in the spectrum are due to exchange rather than TOCSY type transfer as they are also present in EXSY spectra (Figure S14 and S15).


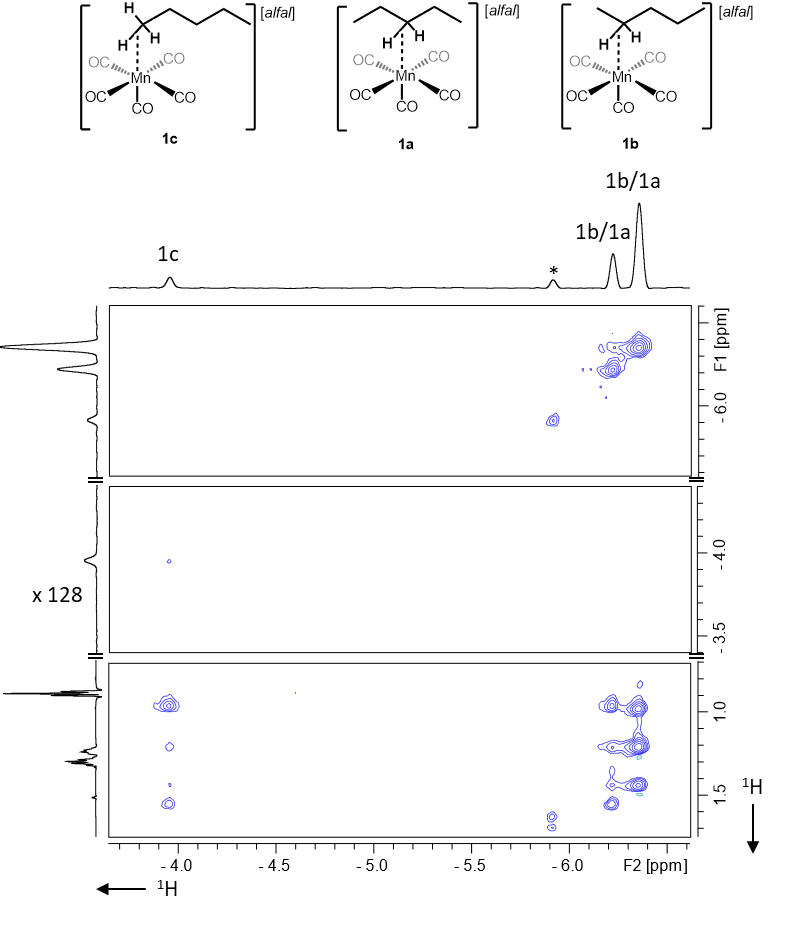


Figure S13 The high field region of a 700 MHz 2D ^1^H-^1^H TOCSY NMR spectrum of the three isomers of [Mn(CO)_5_(*n*‑pentane)]^+^ (**1a-c**) generated by oxidation of Mn_2_(CO)_10_ by [C_10_F_8_]^+.^[*alfal*]^−^ in the presence of *n*-pentane in, collected at −90 ℃ in HFP solvent with a TOCSY mixing time of 50 ms.*denotes a the complex [Mn(CO)_5_(*c*‑pentane)]^+^ formed as a result of contamination of *n*-pentane with *c*-pentane (*ca* 4% mol/mol). Some cross peaks in the spectrum are due to exchange rather than TOCSY type transfer as they are also present in EXSY spectra (Figure S14 and S15).


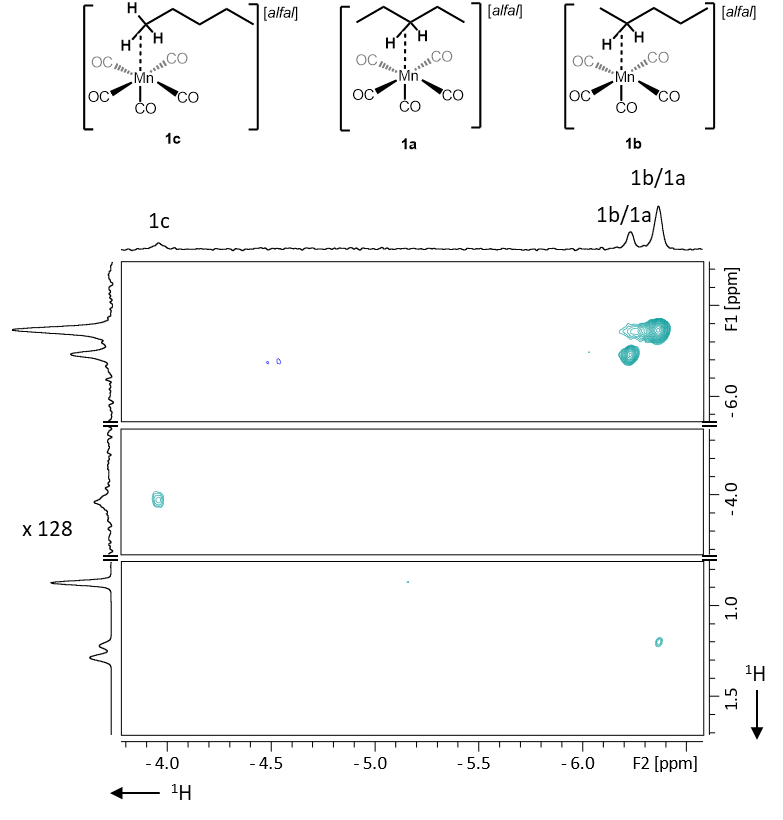


Figure S14 Expansion of the high field region of the 700 MHz ^1^H–^1^H 2D EXSY spectrum (10 ms mixing time) of the three isomers of [Mn(CO)_5_(*n*‑pentane)]^+^ (**1a-c**) collected at −90 ℃ in HFP solvent.


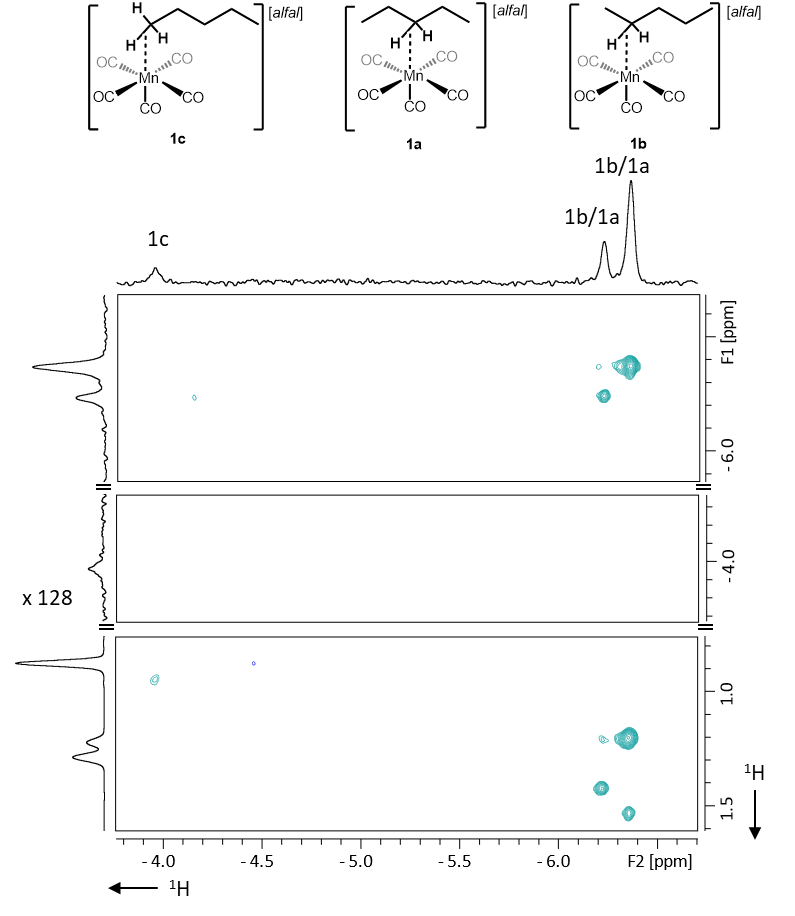


Figure S15 Expansion of the high field region of the 700 MHz ^1^H–^1^H 2D EXSY spectrum (200 ms mixing time) of the three isomers of [Mn(CO)_5_(*n*‑pentane)]^+^ (**1a-c**) collected at −90 ℃ in HFP solvent.


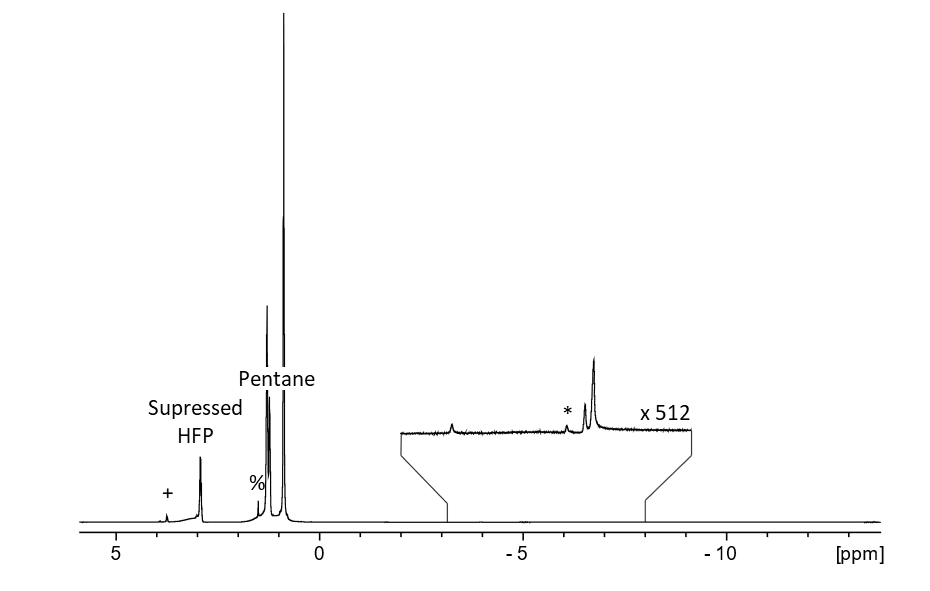


Figure S16 The full 700 MHz ^1^H NMR spectrum of the three isomers of [Mn(CO)_5_(*n*‑pentane)]^+^ (**1a-c**) in an *n*-pentane/HFP mixed solvent system, collected at −90 °C in HFP solvent.*denotes a the complex [Mn(CO)_5_(*c*‑pentane)]^+^ formed as a result of contamination of *n*-pentane with *c*-pentane (*ca* 4% mol/mol, marked with the symbol %). + denotes the minor impurity CF_3_CH_2_Cl found in the solvent

# DFT Calculations

## EDA-NOCV Results

Table S2: EDA-NOCV results of the isoelectronic series of M(CO)_5_(*n*-pentane) complexes in kJ mol^−1^.

|  | C3-Cr(CO)_5_  (*n*‑pentane) | C1-[Mn(CO)_5_  (*n‑*pentane)]^+^  (**1c**) | C2-[Mn(CO)_5_  (*n‑*pentane)]^+^  (**1b**) | C3-[Mn(CO)_5_  (*n‑*pentane)]^+^  (**1a**) |
| --- | --- | --- | --- | --- |
| Δ*E*_int_ | −65.0 | −102.4 | −114.9 | −116.8 |
| Δ*E*_Pauli_ | +94.1 | +103.6 | +107.8 | +115.7 |
| Δ*E*_disp_ | −45.6 | −38.1 | −45.8 | −47.4^a^ |
| Δ*E*_elstat_ | −53.4 | −54.2 | −53.7 | −61.0 |
| Δ*E*_orb_ | −60.1 | −113.7 | −123.2 | −124.2 |
| Δ*E*_orb(1)_ | −41.6 (69 %) | −83.9 (74 %) | −92.0 (75 %) | −90.5 (73 %) |
| Δ*E*_orb(2+3)_ | −10.1 (17 %) | −12.9 (11 %) | −11.8 (9.6 %) | −12.7 (10 %) |

a – for comparison with literature studies,^[19]^ the Δ*E*_disp_ was also calculated using the LED method implemented in Orca 6.0.1 at the DLPNO-CCSD(T)/def2-TZVPP level which leads to Δ*E*_disp_ = –57.7 kJ mol^–1^ if the effect of triples is not included, or Δ*E*_disp_ = –68.4 kJ mol^–1^ if the dispersion component of triples is estimated.

## AIM Analysis


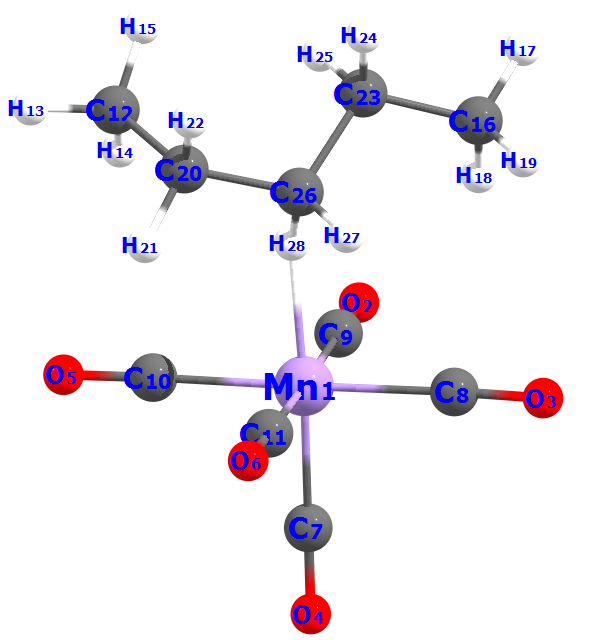
Table S3: Properties of the bond critical points (BCPs) in the MN15/def2-TZVPP optimized structure of the C3-bound isomer of [Mn(CO)_5_(*n*-pentane)]^+^ (**1a**).

| #BCP | Atoms | *ρ* (r_c_) in *e*^−^ Å^−3^ | ∇^2^*ρ* (r_c_) in *e*^−^ Å^−5^ |
| --- | --- | --- | --- |
| **BCP1** | **mn1 - h28** | **0.3278766** | **4.8247136** |
| BCP2 | mn1 - c9 | 0.74477777 | 12.9475423 |
| BCP3 | mn1 - c8 | 0.73990544 | 12.9400716 |
| BCP4 | mn1 - c7 | 0.84741382 | 14.4520661 |
| BCP5 | o4 - c7 | 3.36075874 | 17.552931 |
| BCP6 | mn1 - c10 | 0.7448655 | 13.0098141 |
| BCP7 | mn1 - c11 | 0.7442514 | 12.9430117 |
| BCP8 | o3 - c8 | 3.38310261 | 18.4823084 |
| BCP9 | o2 - c9 | 3.3823198 | 18.4610049 |
| BCP10 | o5 - c10 | 3.38169895 | 18.424495 |
| BCP11 | o6 - c11 | 3.38134803 | 18.4225911 |
| BCP12 | o5 - h14 | 0.03791912 | 0.45361523 |
| BCP13 | c12 - c20 | 1.66241519 | -14.6790545 |
| BCP14 | c12 - h13 | 1.91450753 | -24.2746446 |
| BCP15 | c12 - h14 | 1.8622481 | -22.8822774 |
| BCP16 | c12 - h15 | 1.91061371 | -24.1352078 |
| BCP17 | c8 - h18 | 0.04391843 | 0.47320771 |
| BCP18 | c16 - c23 | 1.67605366 | -14.8954875 |
| BCP19 | c16 - h17 | 1.91701792 | -24.3712092 |
| BCP20 | c16 - h18 | 1.86114137 | -22.8499125 |
| BCP21 | c16 - h19 | 1.89023362 | -23.6255866 |
| **BCP22** | **c26 - h28** | **1.53152369** | **-15.6461709** |
| BCP23 | c23 - c26 | 1.66699059 | -14.653365 |
| BCP24 | c20 - h21 | 1.88735881 | -23.4736906 |
| BCP25 | c20 - c26 | 1.6769242 | -14.902187 |
| BCP26 | c20 - h22 | 1.90487085 | -24.0124235 |
| BCP27 | c23 - h24 | 1.89272377 | -23.7111862 |
| BCP28 | c23 - h25 | 1.91597192 | -24.2404481 |
| **BCP29** | **c26 - h27** | **1.92633068** | **-24.6526372** |


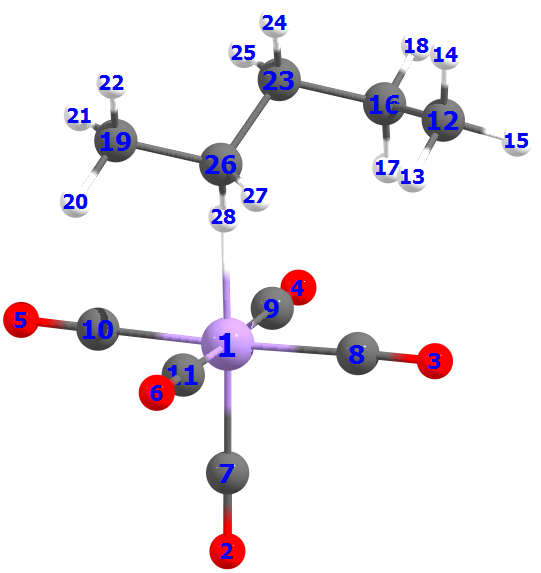
Table S4: Properties of the bond critical points (BCPs) in the MN15/def2-TZVPP optimized structure of the C2-bound isomer of [Mn(CO)_5_(*n*-pentane)]^+^ (**1b**).

| #BCP | Atoms | *ρ* (r_c_) in *e*^−^ Å^−3^ | ∇^2^*ρ* (r_c_) in *e*^−^ Å^−5^ |
| --- | --- | --- | --- |
| BCP1 | mn1 - c7 | 0.84542305 | 14.4400648 |
| BCP2 | o2 - c7 | 3.36130536 | 17.5699449 |
| BCP3 | mn1 - c8 | 0.73939257 | 12.9232023 |
| BCP4 | mn1 - c9 | 0.74580352 | 12.9747982 |
| **BCP5** | **mn1 - h28** | **0.3384648** | **5.04266484** |
| BCP6 | mn1 - c10 | 0.74604646 | 12.8798964 |
| BCP7 | mn1 - c11 | 0.74544586 | 12.95576 |
| BCP8 | o3 - c8 | 3.38201612 | 18.4714398 |
| BCP9 | o4 - c9 | 3.38274495 | 18.5073232 |
| BCP10 | o5 - c10 | 3.38325782 | 18.5101428 |
| BCP11 | o6 - c11 | 3.38115908 | 18.40283 |
| BCP12 | o3 - h13 | 0.03446396 | 0.49311347 |
| BCP13 | c12 - h13 | 1.87475284 | -23.1279907 |
| BCP14 | c12 - c16 | 1.66178084 | -14.6615586 |
| BCP15 | c12 - h14 | 1.8996746 | -23.851635 |
| BCP16 | c12 - h15 | 1.90742848 | -24.0827925 |
| BCP17 | c8 - h17 | 0.04524111 | 0.45409721 |
| BCP18 | c16 - h17 | 1.84519496 | -22.4282525 |
| BCP19 | c16 - c23 | 1.67224758 | -14.8155753 |
| BCP20 | c16 - h18 | 1.92060806 | -24.3408686 |
| BCP21 | c23 - c26 | 1.6621655 | -14.5556195 |
| BCP22 | c19 - h20 | 1.89187347 | -23.6632774 |
| **BCP23** | **c26 - h28** | **1.53860949** | **-15.7915842** |
| BCP24 | c19 - h21 | 1.90082182 | -23.9222451 |
| BCP25 | c19 - c26 | 1.68620997 | -15.0940391 |
| BCP26 | c19 - h22 | 1.90531624 | -24.1065782 |
| BCP27 | c23 - h24 | 1.88752752 | -23.6029576 |
| BCP28 | c23 - h25 | 1.90501931 | -23.9413797 |
| **BCP29** | **c26 - h27** | **1.94025933** | **-25.0564157** |


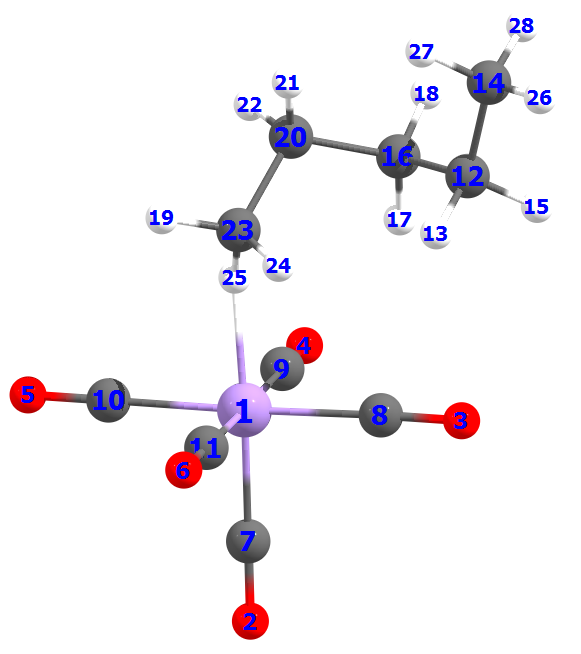
Table S5: Properties of the bond critical points (BCPs) in the MN15/def2-TZVPP optimized structure of the C1-bound isomer of [Mn(CO)_5_(*n*-pentane)]^+^ (**1a**).

| #BCP | Atoms | *ρ* (r_c_) in *e*^−^ Å^−3^ | ∇^2^*ρ* (r_c_) in *e*^−^ Å^−5^ |
| --- | --- | --- | --- |
| BCP1 | mn1 - c8 | 0.73435828 | 12.8889576 |
| BCP2 | mn1 - c7 | 0.84800093 | 14.4769604 |
| BCP3 | o2 - c7 | 3.36206117 | 17.6180465 |
| BCP4 | mn1 - c9 | 0.7419502 | 13.0011625 |
| **BCP5** | **mn1 - h25** | **0.32004848** | **4.61753461** |
| BCP6 | mn1 - c10 | 0.74412993 | 12.9163582 |
| BCP7 | mn1 - c11 | 0.74060727 | 12.9257809 |
| BCP8 | o3 - c8 | 3.38380444 | 18.5971642 |
| BCP9 | o4 - c9 | 3.38418235 | 18.6016707 |
| BCP10 | o5 - c10 | 3.38580871 | 18.684523 |
| BCP11 | o6 - c11 | 3.38478296 | 18.614636 |
| BCP12 | o3 - h13 | 0.03519278 | 0.50340374 |
| BCP13 | c12 - h13 | 1.8680787 | -22.9566469 |
| BCP14 | c12 - c14 | 1.66035694 | -14.6197469 |
| BCP15 | c12 - h15 | 1.90595059 | -23.9370178 |
| BCP16 | c12 - c16 | 1.65687478 | -14.5468957 |
| BCP17 | c14 - h28 | 1.89617894 | -23.770807 |
| BCP18 | c16 - h17 | 1.84059256 | -22.3118303 |
| BCP19 | c16 - h18 | 1.90926404 | -24.0789126 |
| **BCP20** | **c23 - h25** | **1.58916157** | **-16.7767267** |
| BCP21 | c20 - c23 | 1.6422443 | -14.2843131 |
| BCP22 | c16 - c20 | 1.66682188 | -14.717396 |
| BCP23 | c20 - h21 | 1.90225248 | -23.9775764 |
| BCP24 | c20 - h22 | 1.91803018 | -24.2510276 |
| **BCP25** | **h19 - c23** | **1.93465818** | **-24.9331976** |
| **BCP26** | **c23 - h24** | **1.93365942** | **-24.921847** |
| BCP27 | c14 - h26 | 1.90828552 | -24.0897571 |
| BCP28 | c14 - h27 | 1.88781095 | -23.498609 |
| BCP1 | mn1 - c8 | 0.73435828 | 12.8889576 |


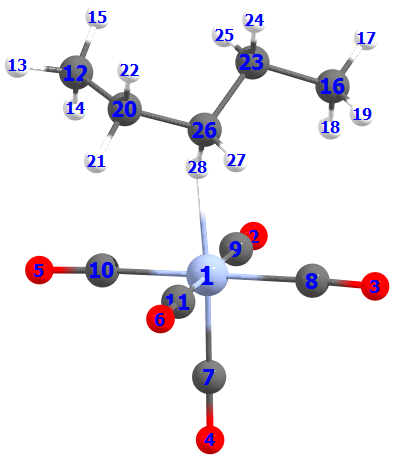
Table S6: Properties of the bond critical points (BCPs) in the MN15/def2-TZVPP optimized structure of the C3-bound isomer of Cr(CO)_5_(*n*-pentane).

| #BCP | Atoms | *ρ* (r_c_) in *e*^−^ Å^−3^ | ∇^2^*ρ* (r_c_) in *e*^−^ Å^−5^ |
| --- | --- | --- | --- |
| **BCP1** | **cr1 - h28** | **0.23945263** | **3.60075015** |
| BCP2 | cr1 - c9 | 0.73330553 | 13.4050856 |
| BCP3 | cr1 - c8 | 0.72912154 | 13.309003 |
| BCP4 | cr1 - c7 | 0.8541487 | 14.8825222 |
| BCP5 | o4 - c7 | 3.22517712 | 11.7762592 |
| BCP6 | cr1 - c10 | 0.73523557 | 13.411255 |
| BCP7 | cr1 - c11 | 0.73314357 | 13.3598037 |
| BCP8 | o3 - c8 | 3.26590356 | 13.6221211 |
| BCP9 | o2 - c9 | 3.26381157 | 13.5368589 |
| BCP10 | o5 - c10 | 3.26239441 | 13.4702975 |
| BCP11 | o6 - c11 | 3.26383856 | 13.5877801 |
| BCP12 | c10 - h14 | 0.03405231 | 0.39915152 |
| BCP13 | c12 - c20 | 1.66249617 | -14.6807896 |
| BCP14 | c12 - h13 | 1.89954638 | -23.8164746 |
| BCP15 | c12 - h14 | 1.87706754 | -23.2651862 |
| BCP16 | c12 - h15 | 1.89542312 | -23.6972569 |
| BCP17 | c16 - c23 | 1.67015558 | -14.7985132 |
| BCP18 | c16 - h17 | 1.89934393 | -23.8300424 |
| BCP19 | c16 - h18 | 1.88024602 | -23.3507376 |
| BCP20 | c16 - h19 | 1.88645453 | -23.5152132 |
| **BCP21** | **c26 - h28** | **1.68253885** | **-18.815501** |
| BCP22 | c23 - c26 | 1.66803659 | -14.674307 |
| BCP23 | c23 - h24 | 1.88509136 | -23.4232997 |
| BCP24 | c20 - h21 | 1.8965231 | -23.7170181 |
| BCP25 | c20 - c26 | 1.67554078 | -14.8791002 |
| BCP26 | c20 - h22 | 1.89053055 | -23.5654355 |
| BCP27 | c23 - h25 | 1.90356166 | -23.8727458 |
| **BCP28** | **c26 - h27** | **1.91763877** | **-24.3377357** |

## NMR Calculations

Note: Calculated absolute shielding of TMS ^1^H atoms = 31.75 ppm; Chemical shift = shielding of TMS proton - shielding of proton

Table S7: Calculated chemical shift of the protons in the optimized structure of the C1-bound isomer of [Mn(CO)_5_(*n*‑pentane)]^+^ (**1c**) using the B3LYP(D3BJ)/def2-TZVPP level of theory and the 5a grid.

|  | **C1** | **C2** | **C3** | **C4** | **C5** |
| --- | --- | --- | --- | --- | --- |
| **Absolute shielding of the protons in ppm** | 30.77 | 29.56 | 30.88 | 30.76 | 30.38 |
|  | 30.15 | 30.31 | 29.74 | 30.00 | 30.86 |
|  | 48.51 |  |  |  | 30.97 |
| **Average absolute shielding of the protons at the C atom in ppm** | 36.48 | 29.93 | 30.31 | 30.38 | 30.73 |
| **Average chemical shifts of the protons at the carbon atom in ppm relative to tetramethylsilane** | -4.76 | 1.78 | 1.40 | 1.33 | 0.98 |

Table S8: Calculated chemical shift of the protons in the optimized structure of the C2-bound isomer of [Mn(CO)_5_(*n*‑pentane)]^+^ (**1b**) using the B3LYP(D3BJ)/def2-TZVPP level of theory and the 5a grid.

|  | **C1** | **C2** | **C3** | **C4** | **C5** |
| --- | --- | --- | --- | --- | --- |
| **Absolute shielding of the protons in ppm** | 30.77 | 29.56 | 30.88 | 30.76 | 30.38 |
|  | 30.15 | 30.31 | 29.74 | 30.00 | 30.86 |
|  | 48.51 |  |  |  | 30.97 |
| **Average absolute shielding of the protons at the C atom in ppm** | 30.41 | 38.90 | 30.35 | 30.35 | 30.70 |
| **Average chemical shifts of the protons at the carbon atom in ppm relative to tetramethylsilane** | 1.31 | -7.19 | 1.37 | 1.37 | 1.01 |

Table S9: Calculated chemical shift of the protons in the optimized structure of the C3-bound isomer of [Mn(CO)_5_(*n*‑pentane)]^+^ (**1a**) using the B3LYP(D3BJ)/def2-TZVPP level of theory and the 5a grid.

|  | **C1** | **C2** | **C3** | **C4** | **C5** |
| --- | --- | --- | --- | --- | --- |
| **Absolute shielding of the protons in ppm** | 30.16 | 30.46 | 29.05 | 30.19 | 29.91 |
|  | 31.32 | 29.85 | 47.60 | 29.92 | 31.36 |
|  | 30.36 |  |  |  | 30.80 |
| **Average absolute shielding of the protons at the C atom in ppm** | 30.61 | 30.15 | 38.33 | 30.05 | 30.69 |
| **Average chemical shifts of the protons at the carbon atom in ppm relative to tetramethylsilane** | 1.10 | 1.56 | **-6.61** | 1.66 | 1.03 |

## B3LYP Optimized Structures

C3-Isomer of [Mn(CO)_5_(*n*-pentane)]^+^ (**1a**) @ B3LYP(D3BJ)/def2-TZVPP


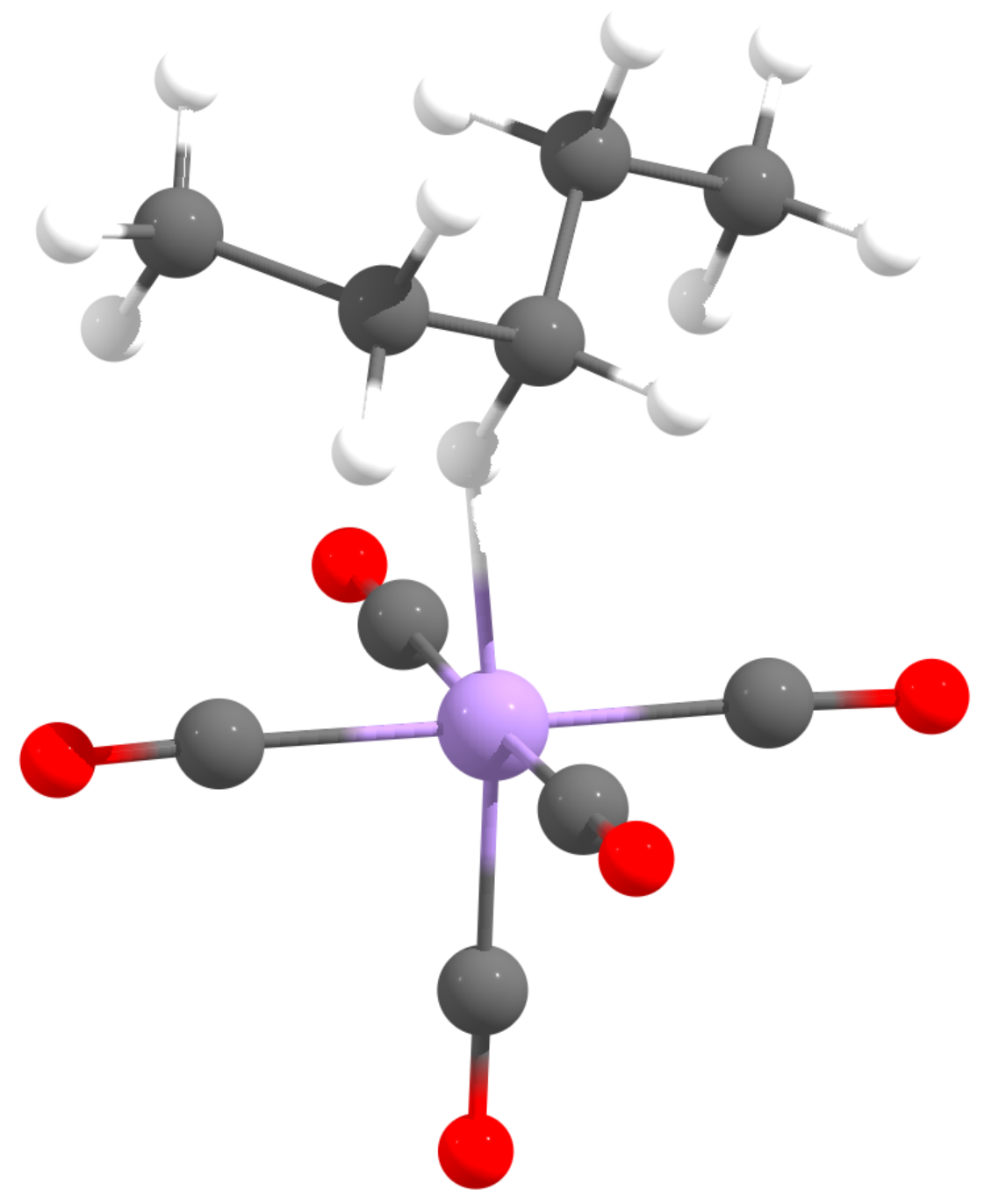


Method: (RI-)B3LYP(D3BJ)/def2-TZVPP

Symmetry: c1

Cartesian coordinates in Ångström:

Mn -0.8820363 -1.2223991 1.0760707

O -0.4715837 0.8567596 3.2484138

O -3.5993566 -0.0527269 0.3927872

O -2.1794368 -3.0950791 3.0054086

O 1.8156721 -2.4578946 1.7079288

O -1.1986621 -3.1864440 -1.2157435

C -1.6903876 -2.3877453 2.2731172

C -2.5799877 -0.4627544 0.6400671

C -0.6274758 0.0849347 2.4428911

C 0.8239423 -1.9812750 1.4667141

C -1.0831746 -2.4591366 -0.3633202

C 2.7380690 0.1638005 -0.6837837

H 3.6152451 -0.3843103 -1.0238509

H 2.6182170 -0.0409762 0.3828067

H 2.9536628 1.2255927 -0.7905711

C -1.2073999 2.4351130 -0.4711928

H -1.2040391 3.5236041 -0.4991338

H -1.4490634 2.1479384 0.5549991

H -2.0135888 2.0913006 -1.1209746

C 1.5049929 -0.2472275 -1.4829633

H 1.4188982 -1.3359227 -1.5072408

H 1.6208930 0.0615652 -2.5250438

C 0.1409398 1.8777952 -0.9063182

H 0.3833695 2.2431759 -1.9087326

H 0.9286934 2.2446083 -0.2481156

C 0.1945935 0.3544621 -0.9819172

H -0.6454695 -0.0140738 -1.5684270

H 0.0744733 0.0173151 0.1061246

SCF energy GEOOPT = -1915.143280149 H

ZPE = 538.6 kJ/mol

FREEH energy = 591.68 kJ/mol

FREEH entropy = 0.62632 kJ/mol/K

C2-Isomer of [Mn(CO)_5_(*n*-pentane)]^+^ (**1b**) @ B3LYP(D3BJ)/def2-TZVPP


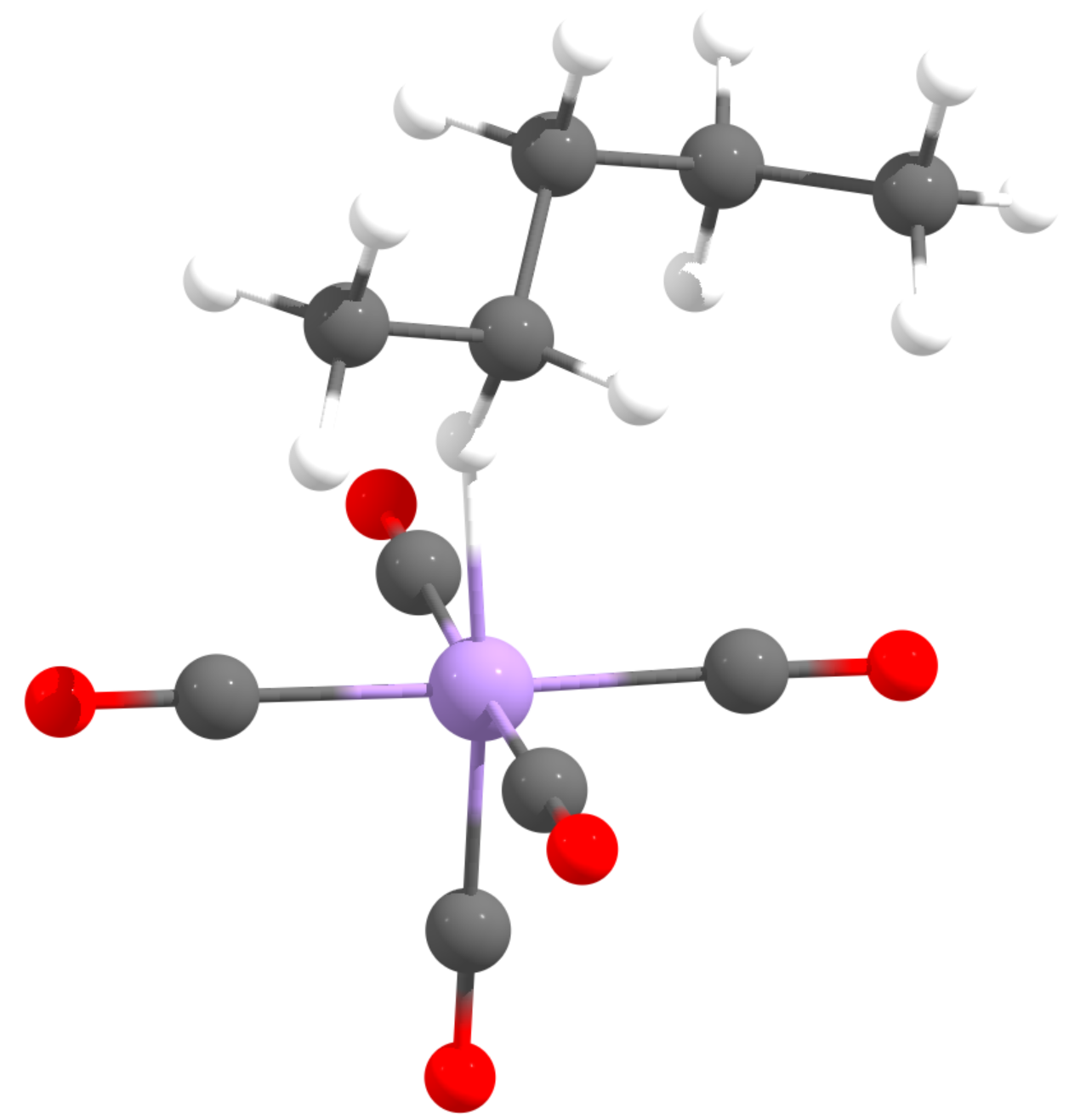


Method: (RI-)B3LYP(D3BJ)/def2-TZVPP

Symmetry: c1

Cartesian coordinates in Ångström:

Mn -1.0897396 1.1299467 -1.1185649

O -2.1032432 3.3310817 -2.8630571

O 1.7613442 2.0800528 -1.5492548

O -1.3155696 2.8827903 1.3484864

O -3.9105448 0.1121483 -0.6516021

O -0.8019562 -0.7609183 -3.4746172

C -1.7182193 2.4978650 -2.2049448

C 0.7106692 1.7145420 -1.3737462

C -1.2289665 2.2327490 0.4326488

C -2.8635678 0.4897669 -0.8240555

C -0.9102797 -0.0554130 -2.6031443

C 2.9085623 -0.2235536 0.7720721

H 2.6537197 -0.3700405 -0.2789859

H 3.3858752 -1.1402209 1.1208331

H 3.6507853 0.5726086 0.8165268

C 1.6923984 0.1118380 1.6324248

H 1.2636983 1.0729499 1.3237878

H 2.0144919 0.2619560 2.6637151

C -1.1731773 -2.2207126 0.2643747

H -1.6531616 -2.3196162 -0.7088168

H -1.9413419 -2.0347554 1.0149946

H -0.7108561 -3.1805265 0.4965338

C 0.6011679 -0.9574453 1.6195019

H 1.0407243 -1.9264831 1.8766256

H -0.1459689 -0.7467938 2.3878740

C -0.1059972 -1.1397992 0.2803398

H 0.6119798 -1.2819672 -0.5237743

H -0.6228270 -0.1320497 0.1238248

SCF energy GEOOPT = -1915.142712534 H

ZPE = 539.1 kJ/mol

FREEH energy = 592.57 kJ/mol

FREEH entropy = 0.63193 kJ/mol/K

C1-Isomer of [Mn(CO)_5_(*n*-pentane)]^+^ (**1c**) @ B3LYP(D3BJ)/def2-TZVPP


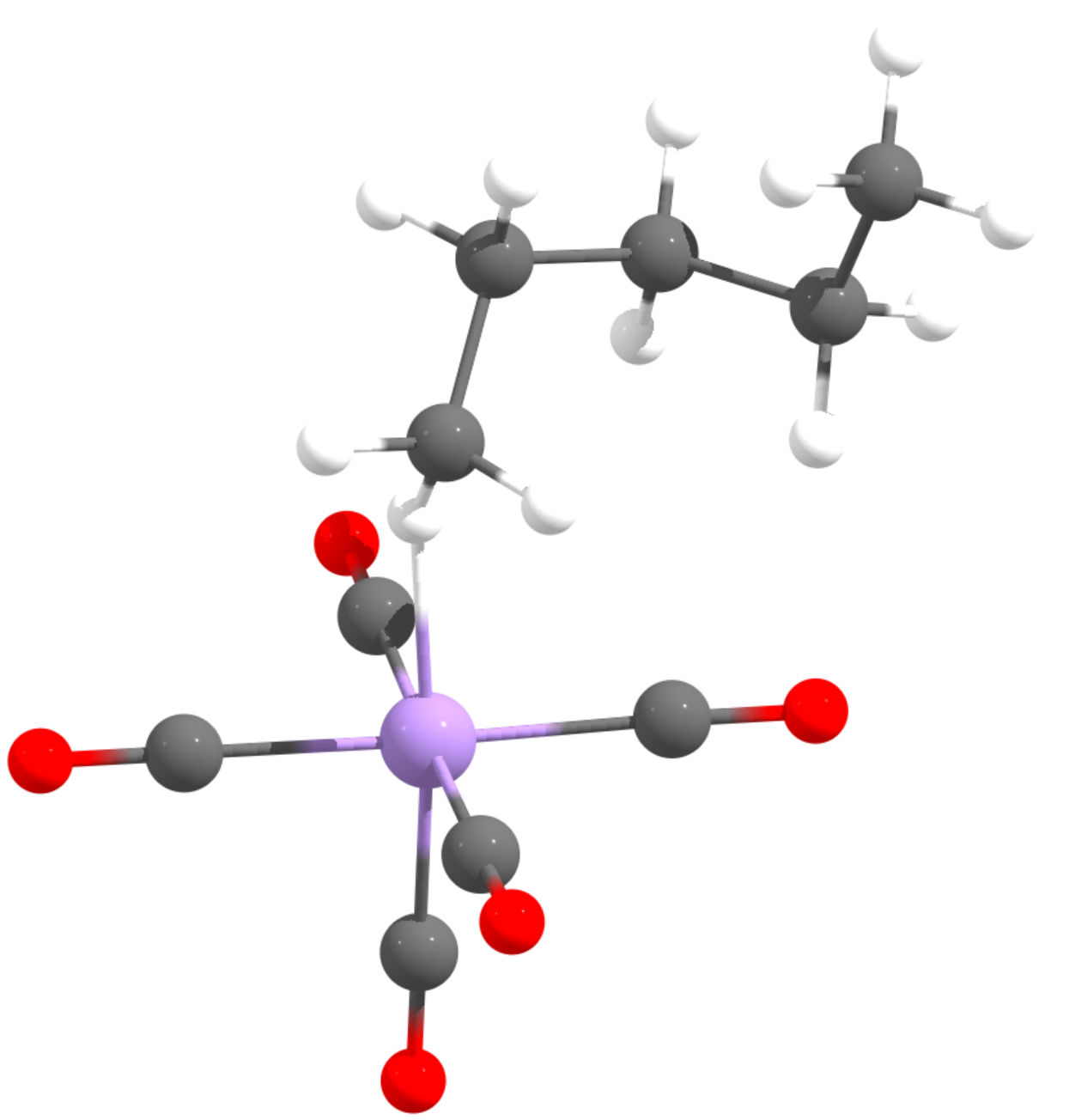


Method: (RI-)B3LYP(D3BJ)/def2-TZVPP

Symmetry: c1

Cartesian coordinates in Ångström:

Mn -1.6620738 1.0458890 -1.2615331

O -2.6575624 3.1509398 -3.1297557

O 1.1779480 2.0657207 -1.6086068

O -2.0243385 2.9063233 1.1103279

O -4.4747694 -0.0294444 -0.8702413

O -1.2397659 -0.9065868 -3.5490241

C -2.2791975 2.3536787 -2.4251901

C 0.1333853 1.6708334 -1.4653156

C -1.8901754 2.2144779 0.2318973

C -3.4319798 0.3702477 -1.0143848

C -1.3971161 -0.1834744 -2.7002087

C 2.3148980 -0.3256737 0.6511053

H 1.9677769 -0.4197086 -0.3829149

C 3.0825271 -1.5874491 1.0403986

H 2.9976289 0.5263223 0.6536370

C 1.1435277 -0.0035601 1.5849284

H 0.7363384 0.9858412 1.3409657

H 1.5208744 0.0832241 2.6057360

H -1.5648040 -1.8082738 0.2511098

C 0.0028518 -1.0214892 1.5762620

H 0.3851740 -2.0205886 1.7983927

H -0.7128003 -0.7857214 2.3652017

C -0.7306203 -1.1110210 0.2423322

H -0.0673754 -1.3485289 -0.5830244

H -1.1879032 -0.0842153 0.1140062

H 3.9405181 -1.7376134 0.3860624

H 2.4635206 -2.4835063 0.9737824

H 3.4535128 -1.5166431 2.0640539

SCF energy GEOOPT = -1915.137647310 H

ZPE = 538.0 kJ/mol

FREEH energy = 589.31 kJ/mol

FREEH entropy = 0.62133 kJ/mol/K

[Mn(CO)_5_]^+^ @ B3LYP(D3BJ)/def2-TZVPP


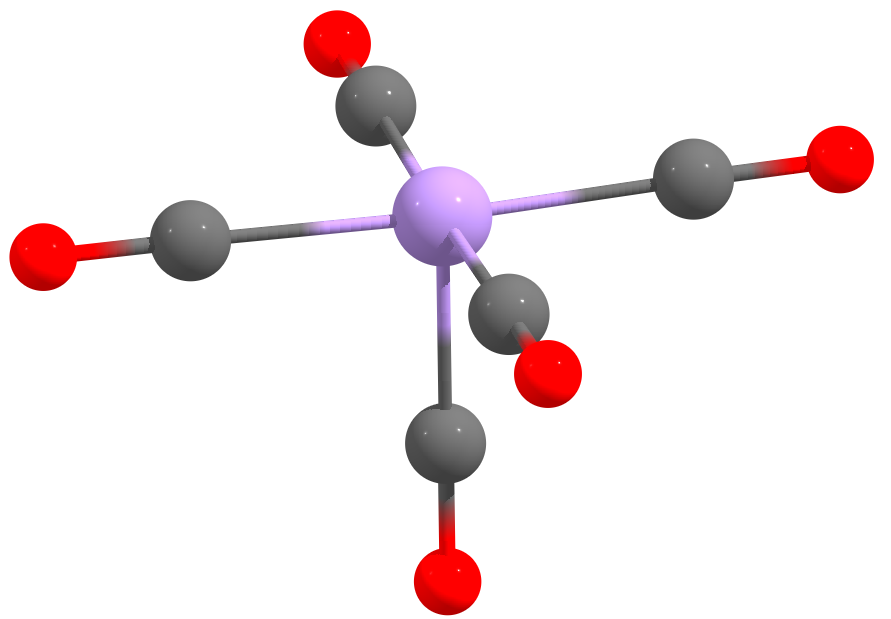


Method: (RI-)B3LYP(D3BJ)/def2-TZVPP

Symmetry: c4v

Cartesian coordinates i

n Ångström:

Mn -0.0000000 0.0000000 0.3939888

O 2.1554327 2.1554327 0.4601597

O 2.1554327 -2.1554327 0.4601597

O 0.0000000 0.0000000 -2.5840764

O -2.1554327 2.1554327 0.4601597

O -2.1554327 -2.1554327 0.4601597

C 0.0000000 0.0000000 -1.4558984

C 1.3605961 -1.3605961 0.4470030

C 1.3605961 1.3605961 0.4470030

C -1.3605961 1.3605961 0.4470030

C -1.3605961 -1.3605961 0.4470030

SCF energy GEOOPT = -1717.385337815 H

ZPE = 110.6 kJ/mol

FREEH energy = 142.54 kJ/mol

FREEH entropy = 0.45238 kJ/mol/K

*n*-Pentane @ B3LYP(D3BJ)/def2-TZVPP


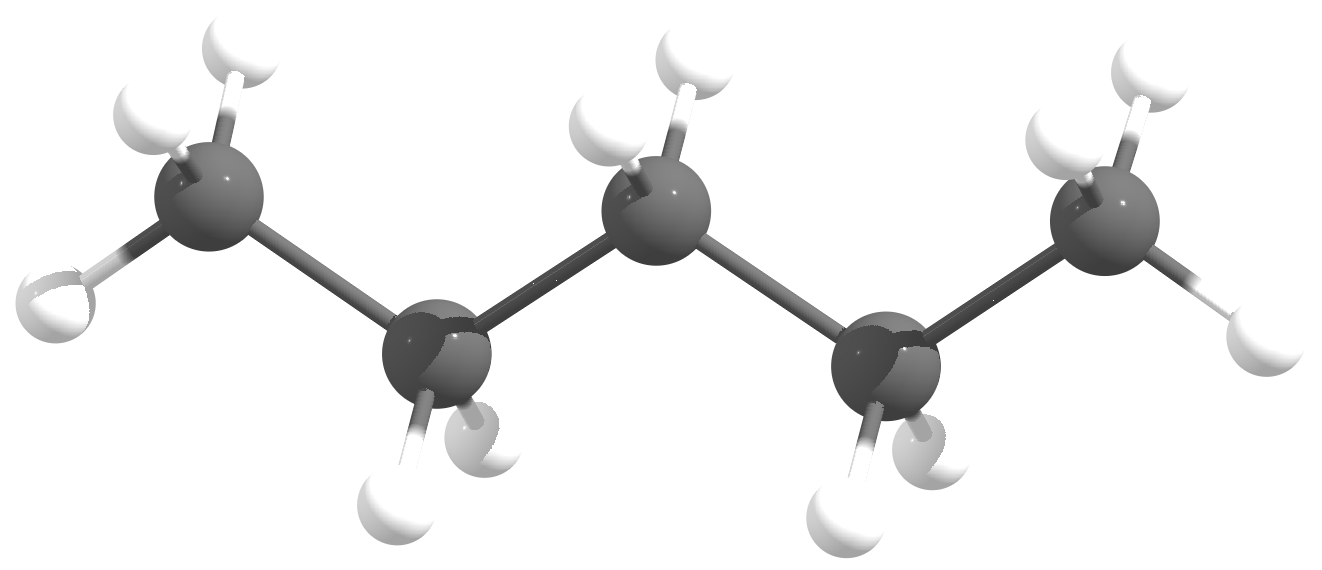


Method: (RI-)B3LYP(D3BJ)/def2-TZVPP

Symmetry: c1

Cartesian coordinates in Ångström:

C -2.4615042 0.7229505 0.0097278

C -0.9623038 0.9990749 0.0908429

H -3.0444049 1.6405822 0.1021976

H -2.7792241 0.0454218 0.8052837

H -2.7235770 0.2569144 -0.9426911

C -0.1126169 -0.2634031 -0.0358245

H -0.7315344 1.4934533 1.0395066

H -0.6764664 1.7046133 -0.6954261

C 1.3894256 -0.0002567 0.0447270

H -0.3420244 -0.7592428 -0.9854569

H -0.3978964 -0.9712031 0.7502345

C 2.2277583 -1.2695266 -0.0838408

H 1.6168631 0.4945943 0.9939788

H 1.6726186 0.7071945 -0.7407428

H 1.9862285 -1.9811622 0.7087600

H 3.2954613 -1.0535674 -0.0224978

H 2.0431971 -1.7664373 -1.0387787

SCF energy GEOOPT = -197.7198838046 H

ZPE = 420.1 kJ/mol

FREEH energy = 438.65 kJ/mol

FREEH entropy = 0.33794 kJ/mol/K

[Mn(CO)_5_(N_2_)]^+^ @ B3LYP(D3BJ)/def2-TZVPP


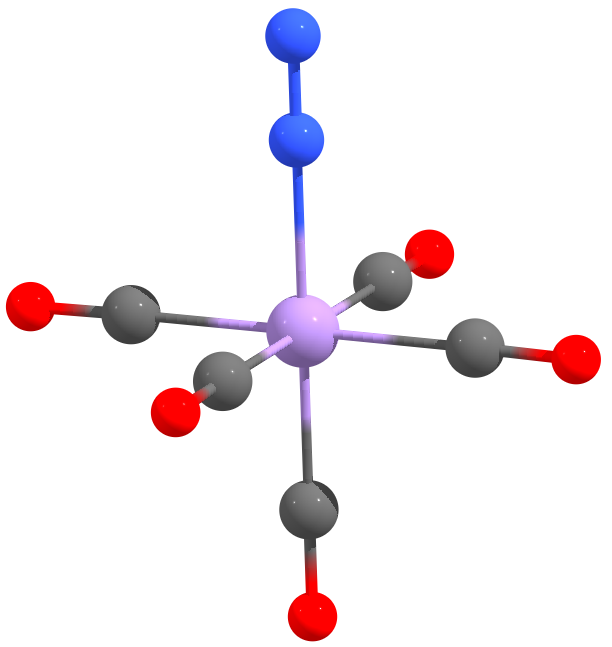


Method: (RI-)B3LYP(D3BJ)/def2-TZVPP

Symmetry: c4v

Cartesian coordinates in Ångström:

Mn -0.0000000 0.0000000 0.0014052

C -1.3578947 1.3578947 0.0038874

C -1.3578947 -1.3578947 0.0038874

C 1.3578947 -1.3578947 0.0038874

C 1.3578947 1.3578947 0.0038874

O -2.1530498 2.1530498 0.0219989

O 2.1530498 2.1530498 0.0219989

O 2.1530498 -2.1530498 0.0219989

O -2.1530498 -2.1530498 0.0219989

C -0.0000000 0.0000000 1.8832482

O 0.0000000 0.0000000 3.0101257

N 0.0000000 0.0000000 -2.0097501

N 0.0000000 0.0000000 -3.1027936

SCF energy GEOOPT = -1826.940423353 H

ZPE = 133.7 kJ/mol

FREEH energy = 172.02 kJ/mol

FREEH entropy = 0.49884 kJ/mol/K

## 7.5 MN15 Optimized Structures

C3-Isomer of [Mn(CO)_5_(*n*-pentane)]^+^ (**1a**) @ MN15/def2-TZVPP


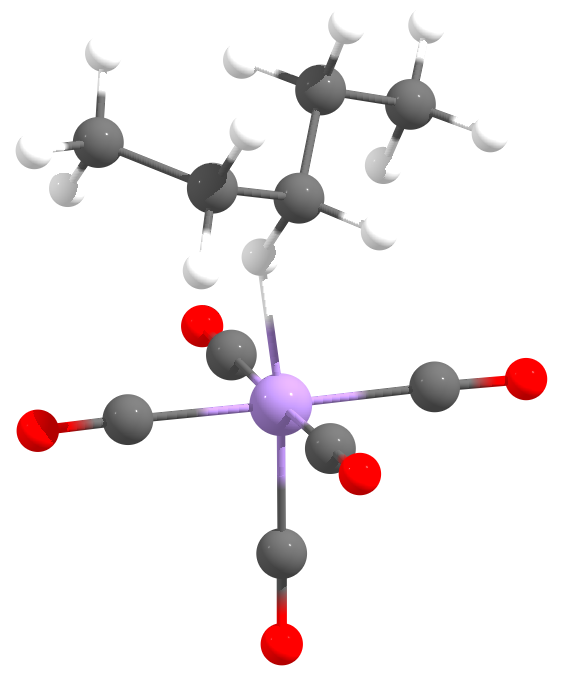


Method: (RI-)mn15/def2-TZVPP

Symmetry: c1

Cartesian coordinates in Ångström:

Mn -0.8568631 -1.1883404 1.0501949

O -0.5412056 0.8198886 3.2989667

O -3.5696062 -0.0316606 0.3391146

O -2.1536058 -3.1214749 2.9132039

O 1.8543583 -2.3909234 1.6742243

O -1.1213020 -3.1185450 -1.2725747

C -1.6645408 -2.3903491 2.2039439

C -2.5529630 -0.4419421 0.6016007

C -0.6576707 0.0751267 2.4606561

C 0.8534045 -1.9268801 1.4418897

C -1.0243219 -2.4036392 -0.4061438

C 2.6925796 0.1691101 -0.6892471

H 3.5620375 -0.4293839 -0.9525983

H 2.5533795 0.0947271 0.3936660

H 2.9271574 1.2078175 -0.9155077

C -1.1727240 2.4310309 -0.4485250

H -1.1710112 3.5180737 -0.4879713

H -1.3790872 2.1507863 0.5889688

H -1.9987255 2.0775921 -1.0685101

C 1.4557190 -0.3011122 -1.4425083

H 1.3732911 -1.3908189 -1.3946064

H 1.5479367 -0.0556262 -2.5029198

C 0.1535597 1.8577955 -0.9156415

H 0.3707731 2.1861567 -1.9360907

H 0.9676063 2.2262923 -0.2899722

C 0.1681655 0.3345056 -0.9351046

H -0.7003151 -0.0272449 -1.4879594

H 0.0839740 0.0690379 0.1794514

SCF energy GEOOPT = -1914.790420894 H

ZPE = 536.9 kJ/mol

FREEH energy = 589.90 kJ/mol

FREEH entropy = 0.61979 kJ/mol/K

C2-Isomer of [Mn(CO)_5_(*n*-pentane)]^+^ (**1b**) @ MN15/def2-TZVPP


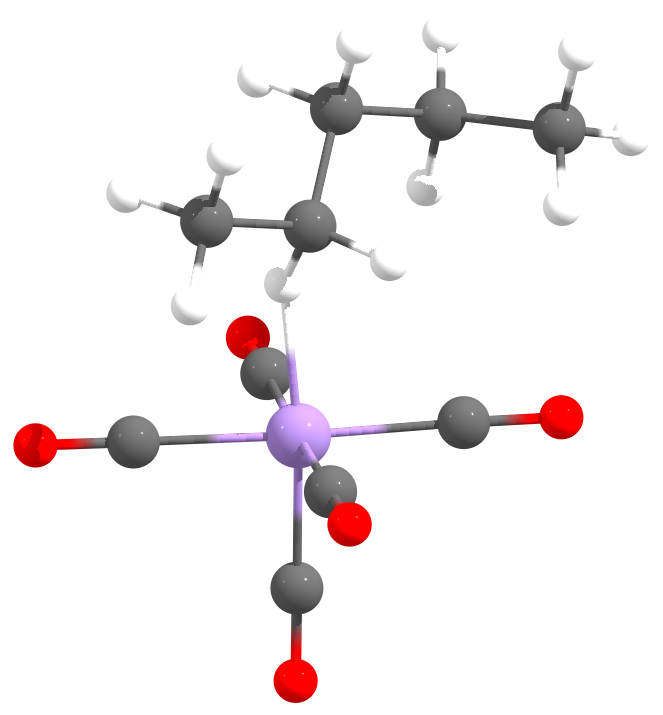


Method: (RI-)mn15/def2-TZVPP

Symmetry: c1

Cartesian coordinates in Ångström:

Mn -1.0755164 1.0948809 -1.0746417

O -2.0771175 3.2383878 -2.8902608

O 1.7818490 2.0084311 -1.5275116

O -1.2884870 2.9654157 1.3006937

O -3.8943403 0.1069186 -0.5605139

O -0.8207771 -0.8587671 -3.3778835

C -1.6954950 2.4250155 -2.2051194

C 0.7261360 1.6556027 -1.3484658

C -1.2069425 2.2669732 0.4195762

C -2.8473851 0.4779842 -0.7526389

C -0.9169631 -0.1282134 -2.5244651

C 2.8913314 -0.2107062 0.7862693

H 2.6329340 -0.3945518 -0.2586744

H 3.3921137 -1.1053614 1.1569236

H 3.6120118 0.6051313 0.8036046

C 1.6717373 0.1046622 1.6426057

H 1.2241475 1.0613674 1.3383312

H 1.9850600 0.2492717 2.6765723

C -1.1746827 -2.1568151 0.1914413

H -1.6654490 -2.2162317 -0.7804784

H -1.9356205 -2.0072092 0.9578267

H -0.7038909 -3.1216044 0.3783012

C 0.6063505 -0.9834336 1.5922649

H 1.0704064 -1.9591059 1.7682511

H -0.1342035 -0.8422693 2.3828243

C -0.1181529 -1.0702285 0.2536681

H 0.5985752 -1.1557253 -0.5623858

H -0.6376292 -0.0498193 0.2138852

SCF energy GEOOPT = -1914.789383409 H

ZPE = 536.3 kJ/mol

FREEH energy = 589.52 kJ/mol

FREEH entropy = 0.62289 kJ/mol/K

C1-Isomer of [Mn(CO)_5_(*n*-pentane)]^+^ (**1c**) @ MN15/def2-TZVPP


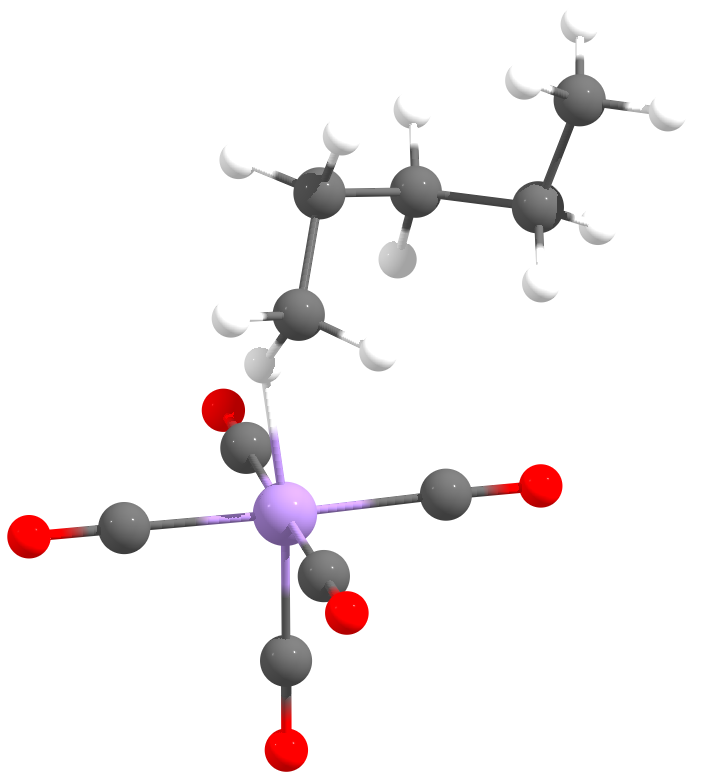


Method: (RI-)mn15/def2-TZVPP

Symmetry: c1

Cartesian coordinates in Ångström:

Mn -1.6636253 1.0213077 -1.2203736

O -2.5820557 3.1898407 -3.0481514

O 1.2234320 1.8429170 -1.6775546

O -1.8154617 2.9323147 1.1294303

O -4.5276369 0.1621349 -0.7213646

O -1.4936537 -0.9382591 -3.5294440

C -2.2321603 2.3664583 -2.3582156

C 0.1585624 1.5208028 -1.4964535

C -1.7554674 2.2154317 0.2617340

C -3.4626390 0.4813946 -0.9050485

C -1.5566231 -0.2098805 -2.6717275

C 2.3002474 -0.3320813 0.6476806

H 1.9995932 -0.5857739 -0.3751318

C 3.1591427 -1.4540229 1.2166024

H 2.8995843 0.5766514 0.5645560

C 1.0731824 -0.0404625 1.5111709

H 0.6258433 0.9234865 1.2278933

H 1.3964218 0.0806703 2.5469712

H -1.5117969 -1.8769883 0.0274111

C -0.0050103 -1.1170782 1.4424447

H 0.4412946 -2.1061540 1.5673680

H -0.7227739 -0.9955574 2.2540806

C -0.7439116 -1.1110014 0.1087953

H -0.0565742 -1.1979110 -0.7290611

H -1.2979264 -0.1216134 0.1271836

H 4.0406740 -1.6261677 0.6018986

H 2.6134081 -2.3967569 1.2740989

H 3.4959301 -1.2037019 2.2232068

SCF energy GEOOPT = -1914.784697445 H

ZPE = 537.4 kJ/mol

FREEH energy = 590.65 kJ/mol

FREEH entropy = 0.63314 kJ/mol/K

[Mn(CO)_5_]^+^ @ MN15/def2-TZVPP


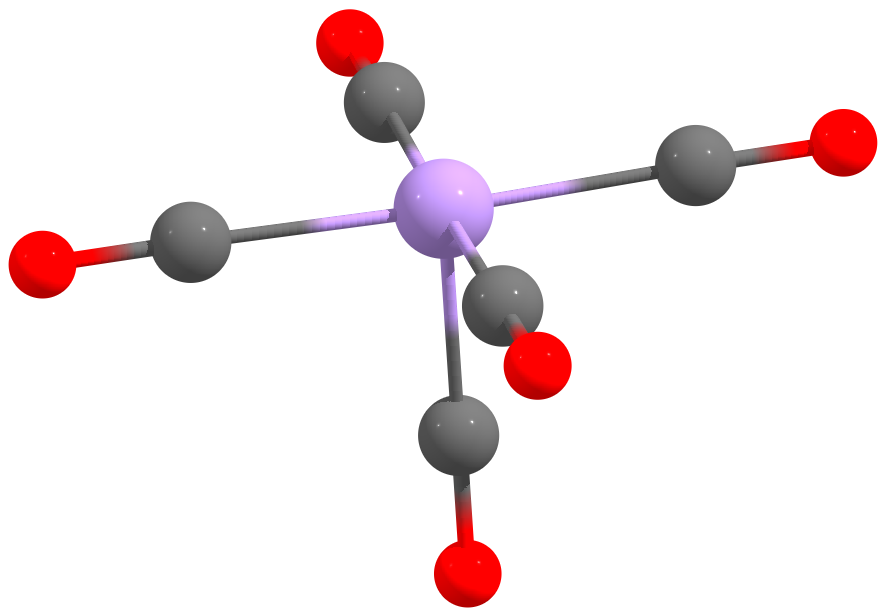


Method: (RI-)mn15/def2-TZVPP

Symmetry: c4v

Cartesian coordinates in Ångström:

Mn -0.0000000 0.0000000 0.4073056

O 2.1522275 2.1522275 0.4486552

O 2.1522275 -2.1522275 0.4486552

O 0.0000000 0.0000000 -2.5671814

O -2.1522275 2.1522275 0.4486552

O -2.1522275 -2.1522275 0.4486552

C 0.0000000 0.0000000 -1.4384472

C 1.3567637 -1.3567637 0.4465917

C 1.3567637 1.3567637 0.4465917

C -1.3567637 1.3567637 0.4465917

C -1.3567637 -1.3567637 0.4465917

SCF energy GEOOPT = -1717.185537418 H

ZPE = 111.6 kJ/mol

FREEH energy = 143.61 kJ/mol

FREEH entropy = 0.45136 kJ/mol/K

*n*-Pentane @ MN15/def2-TZVPP


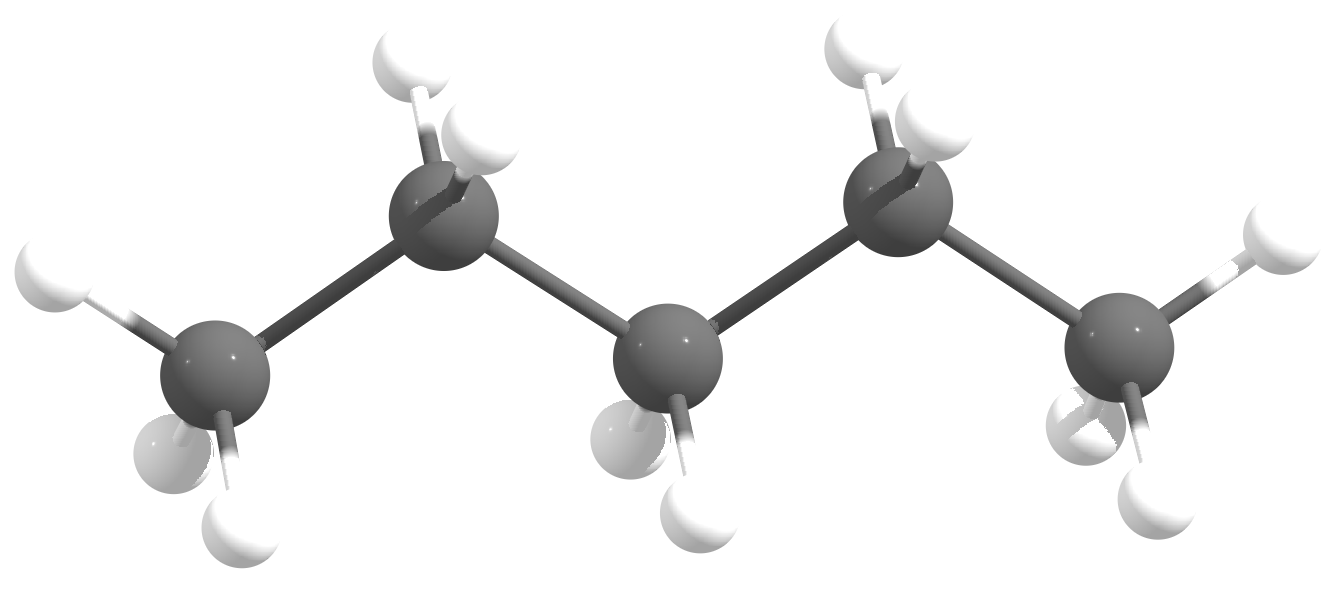


Method: (RI-)mn15/def2-TZVPP

Symmetry: c1

Cartesian coordinates in Ångström:

C -2.4447769 0.7136657 0.0095307

C -0.9528208 1.0018629 0.0914346

H -3.0411699 1.6212104 0.1020955

H -2.7500090 0.0294013 0.8035326

H -2.6955095 0.2433109 -0.9432583

C -0.1109227 -0.2594165 -0.0355366

H -0.7191706 1.4936782 1.0405549

H -0.6645452 1.7052115 -0.6956077

C 1.3848813 0.0085659 0.0448848

H -0.3404625 -0.7552462 -0.9858472

H -0.3959229 -0.9669071 0.7517058

C 2.2094922 -1.2639176 -0.0834997

H 1.6088212 0.5044281 0.9942632

H 1.6645055 0.7156814 -0.7419284

H 1.9553959 -1.9702955 0.7091461

H 3.2793353 -1.0644050 -0.0239333

H 2.0128786 -1.7568284 -1.0375371

SCF energy GEOOPT = -197.5635905034 H

ZPE = 420.9 kJ/mol

FREEH energy = 439.63 kJ/mol

FREEH entropy = 0.33920 kJ/mol/K

C3-Isomer of Cr(CO)_5_(*n*-pentane) @ MN15/def2-TZVPP


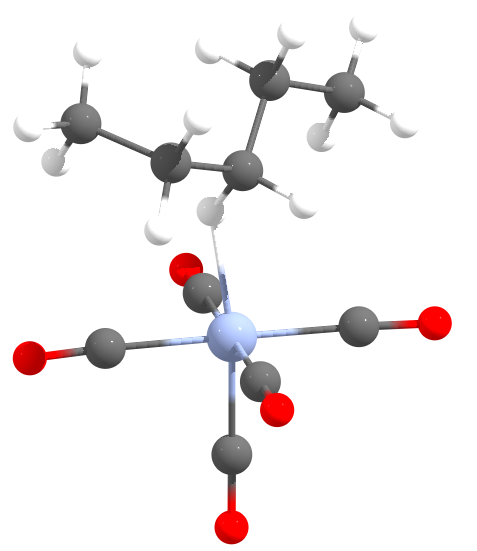


Method: (RI-)mn15/def2-TZVPP

Symmetry: c1

Cartesian coordinates in Ångström:

Cr -0.8801840 -1.2046102 1.0563939

O -0.6520567 0.7610293 3.3789636

O -3.6304592 -0.0784439 0.3552843

O -2.1866339 -3.1437582 2.9282711

O 1.8191436 -2.4333003 1.7692846

O -1.1357657 -3.2284264 -1.2134358

C -1.6887333 -2.4023998 2.2082724

C -2.5912127 -0.4797283 0.6054045

C -0.7244428 0.0424173 2.4943107

C 0.8215360 -1.9510646 1.4918753

C -1.0371492 -2.4605214 -0.3739626

C 2.7292477 0.1661357 -0.7310772

H 3.5965606 -0.3992109 -1.0688530

H 2.6206456 -0.0030507 0.3432062

H 2.9452785 1.2240062 -0.8801987

C -1.1443176 2.4640305 -0.4547514

H -1.1677368 3.5509675 -0.5195056

H -1.2998781 2.1901246 0.5914176

H -1.9885064 2.0734317 -1.0265092

C 1.4699573 -0.2589689 -1.4745270

H 1.3697748 -1.3467190 -1.4373464

H 1.5511371 0.0090505 -2.5316090

C 0.1702896 1.8990950 -0.9689248

H 0.3329728 2.1998606 -2.0083545

H 1.0013869 2.3087874 -0.3912236

C 0.2041637 0.3757822 -0.9111519

H -0.6782451 -0.0143681 -1.4202495

H 0.1732273 0.1398525 0.1889959

SCF energy GEOOPT = -1808.471905067 H

ZPE = 536.0 kJ/mol

FREEH energy = 589.10 kJ/mol

FREEH entropy = 0.63093 kJ/mol/K

α-F Isomer of [Mn(CO)_5_(C_10_F_8_) @ MN15/def2-TZVPP


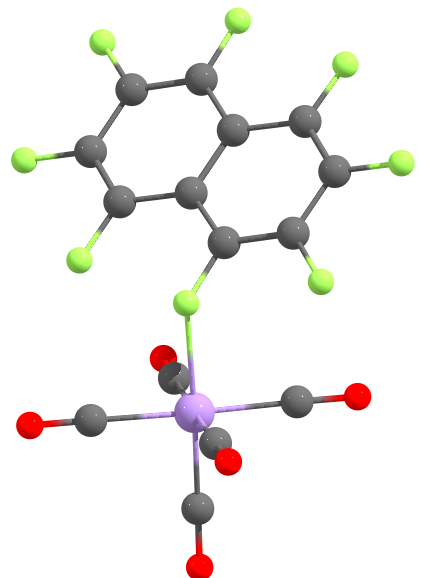


Method: (RI-)mn15/def2-TZVPP

Symmetry: c1

Cartesian coordinates in Ångström:

Mn -0.3619392 0.1964013 2.7102516

C -1.4865595 1.7500970 2.7949296

C -1.6890627 -0.7033430 1.6785488

C 0.7621681 -1.3367067 2.5711646

C 1.0122170 1.1130605 3.6845200

O -2.1534584 2.6550990 2.8502047

O 1.8208969 1.6442531 4.2600199

O 1.4052131 -2.2606266 2.5060284

O -2.4708268 -1.2539832 1.0807548

C -1.0906452 -0.4603797 4.2752069

C 0.5717733 -0.3729575 -2.4431072

O -1.5368373 -0.8587134 5.2344277

C 0.0554785 0.3074876 -1.2989718

C 0.8982629 0.4047018 -0.1770312

C 2.1590376 -0.1112447 -0.1430728

C 2.6638309 -0.7872053 -1.2676842

C 1.8835324 -0.9096441 -2.3880020

F 0.4817633 1.0740546 0.9444433

F 2.9026401 0.0190364 0.9423120

F 3.8773168 -1.2873443 -1.2198556

F 2.3878437 -1.5463400 -3.4206196

C -1.2459815 0.8458565 -1.3522468

C -0.2502851 -0.4786482 -3.5905858

C -2.0147978 0.7278986 -2.4755228

C -1.5101757 0.0582759 -3.6072297

F -1.7526255 1.4806110 -0.2903520

F -3.2267825 1.2292299 -2.5162874

F -2.2704986 -0.0420213 -4.6717005

F 0.1785013 -1.0969053 -4.6705429

SCF energy GEOOPT = -2896.382616594 H

ZPE = 334.2 kJ/mol

FREEH energy = 407.38 kJ/mol

FREEH entropy = 0.78288 kJ/mol/K

F2 Isomer of [Mn(CO)_5_(5FB) @ MN15/def2-TZVPP


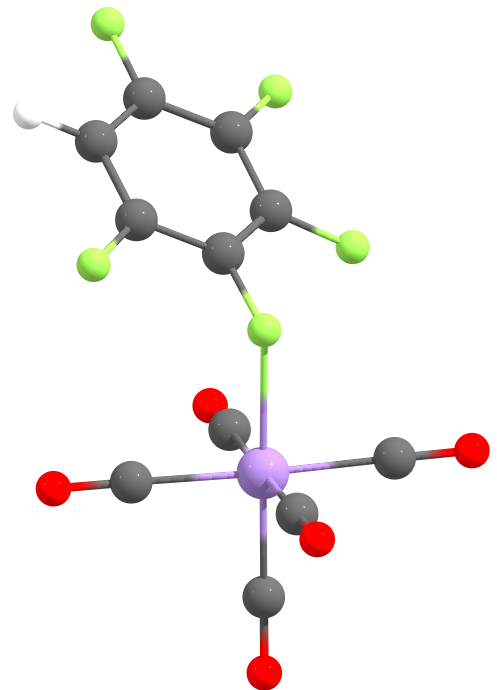


Method: (RI-)mn15/def2-TZVPP

Symmetry: c1

Cartesian coordinates in Ångström:

Mn -0.8822595 0.2807669 1.8666754

C -1.7725466 1.9371871 2.2360679

C -2.4099820 -0.3219704 0.8863534

C 0.0239025 -1.3500958 1.4714573

C 0.6783154 0.9137556 2.7828313

O -2.3023596 2.9029212 2.4685950

O 1.5908952 1.2785217 3.3318915

O 0.5532618 -2.3226514 1.2580869

O -3.3133641 -0.6874715 0.3213497

C -1.5488542 -0.4515097 3.4267678

C 0.1685916 -0.3175154 -3.2128386

O -1.9577597 -0.8977637 4.3813016

C -0.3760423 0.3357756 -2.1241151

C 0.3905841 0.5398660 -0.9931776

C 1.6982986 0.0921935 -0.9151391

C 2.2576308 -0.5668521 -1.9976314

C 1.4793282 -0.7580800 -3.1378516

F -0.1345632 1.2071258 0.0775525

F 2.3801753 0.2839406 0.2036168

F 3.4949601 -1.0040246 -1.9378972

F 2.0223048 -1.3852723 -4.1584792

F -1.6270020 0.7727345 -2.1271711

H -0.4135152 -0.4815816 -4.1082461

SCF energy GEOOPT = -2445.271784484 H

ZPE = 274.3 kJ/mol

FREEH energy = 333.30 kJ/mol

FREEH entropy = 0.68374 kJ/mol/K

[Mn(CO)_5_(HFP) @ MN15/def2-TZVPP


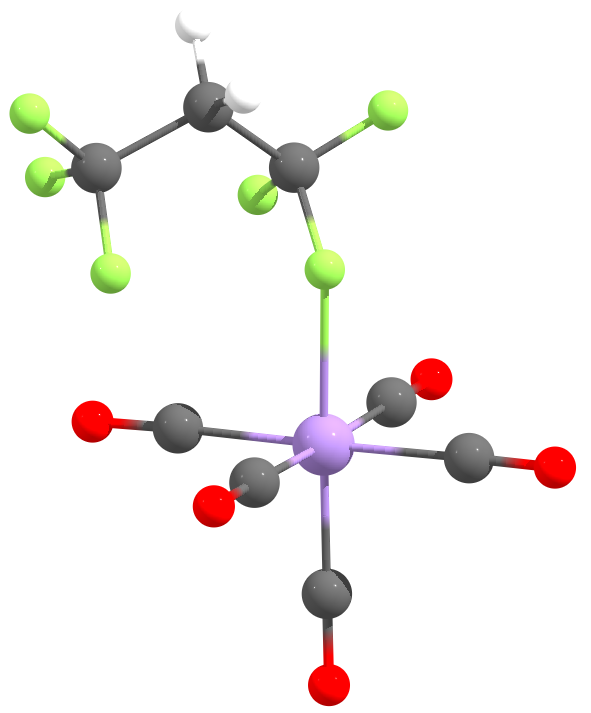


Method: (RI-)mn15/def2-TZVPP

Symmetry: c1

Cartesian coordinates in Ångström:

Mn -0.1735342 -1.3953600 0.9748094

O -0.0745809 0.6210791 3.2495938

O -2.8246647 -0.2188755 0.0608007

O -1.6423535 -3.3239363 2.6991828

O 2.4765934 -2.5664234 1.8901489

O -0.2563493 -3.3728699 -1.3294076

C -1.0847657 -2.5906865 2.0439700

C -1.8320851 -0.6388544 0.3872243

C -0.1067289 -0.1187272 2.4013653

C 1.4997436 -2.1251309 1.5439778

C -0.2195948 -2.6306382 -0.4826453

C -0.4080167 2.6035911 -0.8127018

F -0.3265225 3.9195217 -0.7597868

F -0.5190664 2.1426543 0.4486658

F -1.5243278 2.2666228 -1.4500213

F 1.9445395 0.0842963 -2.1424035

C 0.8258574 2.0362272 -1.4939764

H 0.8353123 2.3769152 -2.5284156

H 1.7137006 2.4140725 -0.9903669

C 0.8872974 0.5309652 -1.5054901

F -0.1941048 -0.0511544 -2.0016972

F 1.0036511 0.0367115 -0.2028265

SCF energy GEOOPT = -2431.538800307 H

ZPE = 268.6 kJ/mol

FREEH energy = 325.48 kJ/mol

FREEH entropy = 0.66563 kJ/mol/K

C_10_F_8_ @ MN15/def2-TZVPP


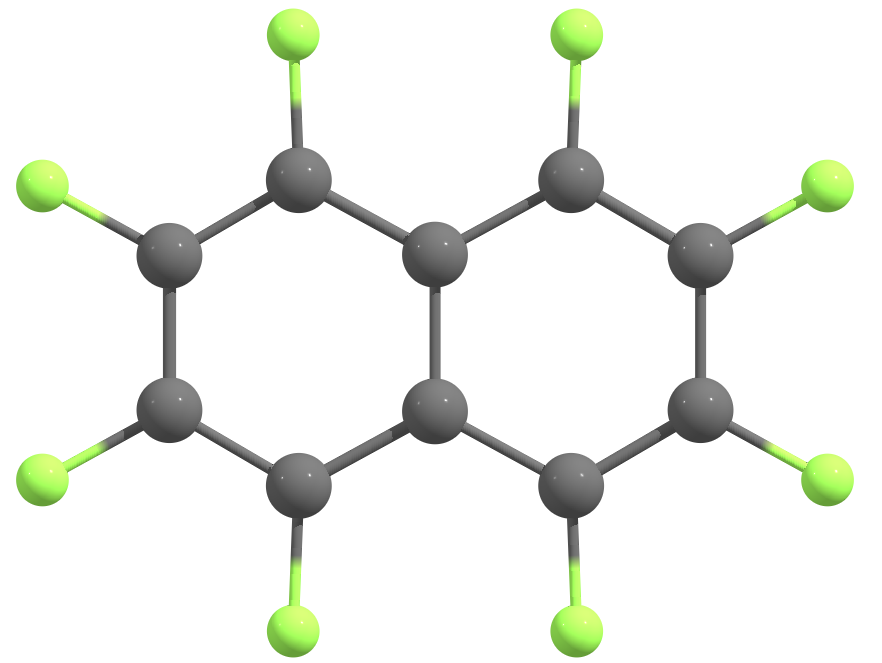


Method: (RI-)mn15/def2-TZVPP

Symmetry: d2h

Cartesian coordinates in Ångström:

C -2.4201008 0.7029828 0.0000000

C -2.4201008 -0.7029828 0.0000000

C -1.2410472 -1.3928412 0.0000000

C 0.0000000 -0.7140888 0.0000000

C 0.0000000 0.7140888 0.0000000

C -1.2410472 1.3928412 0.0000000

C 1.2410472 -1.3928412 0.0000000

C 2.4201008 -0.7029828 0.0000000

C 2.4201008 0.7029828 0.0000000

C 1.2410472 1.3928412 0.0000000

F 1.2919531 2.7168442 0.0000000

F 3.5774290 -1.3395255 0.0000000

F 3.5774290 1.3395255 0.0000000

F 1.2919531 -2.7168442 0.0000000

F -3.5774290 -1.3395255 0.0000000

F -1.2919531 -2.7168442 0.0000000

F -1.2919531 2.7168442 0.0000000

F -3.5774290 1.3395255 0.0000000

SCF energy GEOOPT = -1179.162502179 H

ZPE = 218.6 kJ/mol

FREEH energy = 255.65 kJ/mol

FREEH entropy = 0.48167 kJ/mol/K

5FB @ MN15/def2-TZVPP


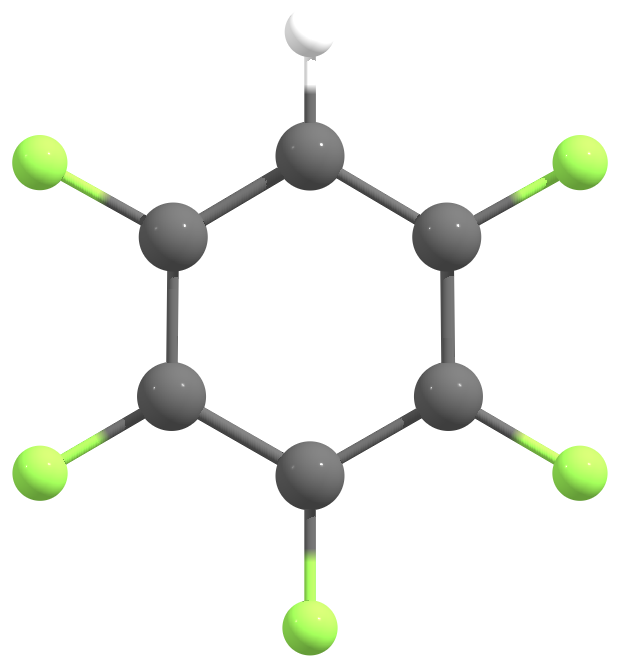


Method: (RI-)mn15/def2-TZVPP

Symmetry: cs

Cartesian coordinates in Ångström:

C -1.4131799 -0.1247967 0.0000000

C -0.7116368 -0.0628918 -1.1891984

C 0.6693649 0.0590560 -1.2039323

C 1.3567047 0.1196687 0.0000000

C 0.6693649 0.0590560 1.2039323

C -0.7116368 -0.0628918 1.1891984

F 1.3343305 0.1176249 -2.3473980

F 2.6734328 0.2355842 0.0000000

F 1.3343305 0.1176249 2.3473980

F -1.3561301 -0.1200622 2.3491335

F -1.3561301 -0.1200622 -2.3491335

H -2.4887613 -0.2199428 0.0000000

SCF energy GEOOPT = -728.0534755572 H

ZPE = 159.1 kJ/mol

FREEH energy = 181.70 kJ/mol

FREEH entropy = 0.38257 kJ/mol/K

HFP @ MN15/def2-TZVPP


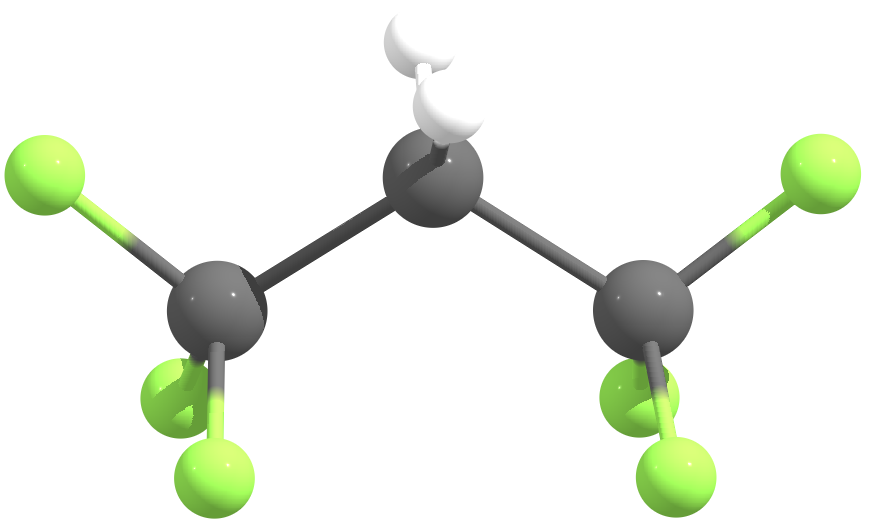


Method: (RI-)mn15/def2-TZVPP

Symmetry: c2v

Cartesian coordinates in Ångström:

C 0.0000000 -1.2771336 0.2074011

C 0.0000000 0.0000000 -0.6061249

F 0.0000000 -2.3249810 -0.6238542

F -1.0765181 -1.3804937 0.9847586

F 1.0765181 -1.3804937 0.9847586

C 0.0000000 1.2771336 0.2074011

H -0.8865075 0.0000000 -1.2367910

H 0.8865075 0.0000000 -1.2367910

F -1.0765181 1.3804937 0.9847586

F 0.0000000 2.3249810 -0.6238542

F 1.0765181 1.3804937 0.9847586

SCF energy GEOOPT = -714.3199402730 H

ZPE = 153.5 kJ/mol

FREEH energy = 172.09 kJ/mol

FREEH entropy = 0.34902 kJ/mol/K

## Calculations of relative energies of conformations of [Mn(CO)_5_(*n*- pentane)]^+^ (**1a-c**)

Table S 10: Calculations of relative energies of conformations of [Mn(CO)_5_(*n*- pentane)]^+^ (**1a-c**).

| ***Isomer*** | **pentane coformation^1^** | **MN15/def2-TZVPP**  **Electronic energy (a.u)^2^** | **MN15/def2-TZVPP**  **Relative electronic energy (kJ/mol)** | **DLPNO-CCSD(T)/def2-TZVPP**  **Electronic energy (a.u)^3^** | **DLPNO-CCSD(T)/def2-TZVPP**  **Relative electronic energy (kJ/mol)** | **Free energy correction term**  **(a.u.) 183K^4^** | **MN15/def2-TZVPP**  **Relative free energy (kJ/mol)** | **DLPNO-CCSD(T)/def2-TZVPP**  **Relative free energy^5^ (kJ/mol)** | **Solvation enegy (kJ/mol)^6^** | **DLPNO-CCSD(T)/def2-TZVPP/SMD**  **Relative free energy^7^ (kJ/mol)** |
| --- | --- | --- | --- | --- | --- | --- | --- | --- | --- | --- |
| ***1c*** *(C1)* | aa | -1914.78549 | 13 | -1913.40511 | 9.4 | 0.180015 | 11.3 | 9.2 | -220.1 | 6.5 |
| ***1c*** *(C1)* | ag | -1914.78566 | 12.6 | -1913.40468 | 10.6 | 0.180607 | 12.4 | 11.9 | -219.7 | 9.6 |
| ***1c*** *(C1)* | gg | -1914.78474 | 15 | -1913.40347 | 13.7 | 0.180472 | 14.5 | 14.7 | -219.9 | 12.2 |
| ***1b*** *(C2)* | ag | -1914.78943 | 2.7 | -1913.40792 | 2.1 | 0.180426 | 2.1 | 2.9 | -217.5 | 2.8 |
| ***1b*** *(C2)* | aa | -1914.78971 | 1.9 | -1913.40871 | 0 | 0.180089 | 0.4 | 0 | -217.4 | 0 |
| ***1b*** *(C2)* | gg | -1914.78758 | 7.5 | -1913.40683 | 4.9 | 0.180297 | 6.6 | 5.5 | -217.2 | 5.6 |
| ***1a*** *(C3)* | ag | -1914.79044 | 0 | -1913.40848 | 0.6 | 0.180652 | 0 | 2.1 | -216.3 | 3.2 |
| ***1a*** *(C3)* | aa | -1914.78946 | 2.6 | -1913.40747 | 3.3 | 0.180386 | 1.9 | 4 | -215.1 | 6.3 |
| ***1a*** *(C3)* | gg | -1914.7891 | 3.5 | -1913.40797 | 1.9 | 0.180268 | 2.5 | 2.4 | -216.4 | 3.3 |

**Notes:**

1. aa = anti-anti; ag = anti-gauche; gg = gauche-gauche. Syn conformer not considered.
2. Geometries optimized using MN15/def2-TZVPP using Orca 6.0.1 with keywords rijcosx, tightscf, verytightopt, defgrid3.
3. Calculated using the MN15/def2-TZVPP geometries using Orca 6.0.1 with keywords rijcosx, verytightscf, tightPNO, defgrid3.
4. Free energy correction term includes zero-point energy, enthalpy correction and entropy terms from MN15/def2-TZVPP frequency calculation at 183 K using quasi-RRHO method.
5. Using the MN15/def2-TZVPP free energy correction term.
6. Solvation energy calculated using the SMD model in HFP solvent with MN15/def2-TZVPP vacuum geometries. Calculations employ M05-2X/6-31+G(d,p) method using Orca 6.0.1 with keywords tightscf, defgrid3. SMD solvent definition in Orca: Epsilon 7.9; Refrac 1.233900; soln 1.233900; soln25 1.230500; sola 0.026700; solb 0.062800; solg 13.800000; solc 0.000000; solh 0.667000.
7. Relative free energy including SMD solvation.

**Comments:** In solution, each of the pentane complexes **1a-c** should exist as a rapidly equilibrating mixture of different conformations of the pentane ligand. Calculations suggest that the C3 isomer **1a** slightly prefers the anti-gauche conformation seen in the crystal structure in vacuum/ HFP solvent as well. However, the C2 isomer **1b** is calculated to prefer the anti-anti conformation in vacuum/solution in contrast to the anti-gauche conformer observed in the solid state. The C1 isomer **1c** also prefers an anti-anti conformation and the relative energy of the C1 isomer is decreased when solvent is included. The electronic energies of the lowest energy conformations of **1a** and **1b** are close, with **1b** preferred over **1a** by 0.5 kJ/mol using the DLPNO-CCSD(T) method. Energy differences between isomers **1a** and **1b** that are slightly larger than small differences observed experimentally are seen when free energies and solvation are employed.

# References

[1] M. Sellin, J. Willrett, D. Röhner, T. Heizmann, J. Fischer, M. Seiler, C. Holzmann, T. A. Engesser, V. Radtke, I. Krossing, *Angew. Chem. Int. Ed.* **2024**, e202406742.

[2] P. J. Malinowski, T. Jaroń, M. Domańska, J. M. Slattery, M. Schmitt, I. Krossing, *Dalton Trans.* **2020**, *49*, 7766.

[3] a) J. D. Watson, L. D. Field, G. E. Ball, *Nat. Chem.* **2022**, *14*, 801; b) J. D. Watson, L. D. Field, G. E. Ball, *J. Am. Chem. Soc.* **2022**, *144*, 17622.

[4] G. M. Sheldrick, *Acta Cryst. A* **2015**, *71*, 3.

[5] G. M. Sheldrick, *Acta Cryst. C* **2015**, *71*, 3.

[6] C. B. Hübschle, G. M. Sheldrick, B. Dittrich, *J. Appl. Cryst.* **2011**, *44*, 1281.

[7] a) D. Kratzert, J. J. Holstein, I. Krossing, *J. Appl. Cryst.* **2015**, *48*, 933; b) D. Kratzert, I. Krossing, *J. Appl. Cryst.* **2018**, *51*, 928.

[8] D. Kratzert, "FinalCif", can be found under https://www.xs3.uni-freiburg.de/research/finalcif.

[9] C. F. Macrae, I. Sovago, S. J. Cottrell, P. T. A. Galek, P. McCabe, E. Pidcock, M. Platings, G. P. Shields, J. S. Stevens, M. Towler et al., *J. Appl. Cryst.* **2020**, *53*, 226.

[10] C. R. Groom, I. J. Bruno, M. P. Lightfoot, S. C. Ward, *Acta Cryst. B* **2016**, *72*, 171.

[11] a) M. von Arnim, R. Ahlrichs, *J. Comput. Chem.* **1998**, *19*, 1746; b) O. Treutler, R. Ahlrichs, *J. Chem. Phys.* **1995**, *102*, 346.

[12] a) A. D. Becke, *J. Chem. Phys.* **1993**, *98*, 1372; b) Lee, Yang, Parr, *Phys. Rev. B Condens. Matter* **1988**, *37*, 785.

[13] H. S. Yu, X. He, S. L. Li, D. G. Truhlar, *Chem. Sci.* **2016**, *7*, 5032.

[14] F. Weigend, R. Ahlrichs, *Phys. Chem. Chem. Phys.* **2005**, *7*, 3297.

[15] a) M. Sierka, A. Hogekamp, R. Ahlrichs, *J. Chem. Phys.* **2003**, *118*, 9136; b) F. Weigend, *Phys. Chem. Chem. Phys.* **2006**, *8*, 1057; c) R. Ahlrichs, *Phys. Chem. Chem. Phys.* **2004**, *6*, 5119.

[16] S. Grimme, S. Ehrlich, L. Goerigk, *J. Comput. Chem.* **2011**, *32*, 1456.

[17] P. Deglmann, F. Furche, R. Ahlrichs, *Chem. Phys. Lett.* **2002**, *362*, 511.

[18] M. K. Assefa, J. L. Devera, A. D. Brathwaite, J. D. Mosley, M. A. Duncan, *Chem. Phys. Lett.* **2015**, *640*, 175.

[19] Q. Lu, F. Neese, G. Bistoni, *Phys. Chem. Chem. Phys.* **2019**, *21*, 11569.
